# Rice growth and yield formation in heterogeneous sodic and saline-sodic soils: challenges and management strategies

**DOI:** 10.3389/fpls.2026.1770941

**Published:** 2026-03-02

**Authors:** Sulaiman Shah, Zijun Cheng, Xinkai Zhang, Yaseen Khan, Zhengwei Liang, Saira Arshad, Qingxue Meng, Peirou Zhen, Zeao Zhang, Tao Zhang, Jialiang Li, Zhonghua Feng, Muhammad Zahid Mumtaz, Mingming Wang

**Affiliations:** 1 Northeast Institute of Geography and Agroecology, Chinese Academy of Sciences, Changchun, China; 2 Key Laboratory of Vegetation Ecology, Ministry of Education, Jilin Songnen Grassland Ecosystem National Observation and Research Station, Northeast Normal University, Changchun, China; 3 Jilin Da’an Farmland Ecosystem National Field Scientific Observation and Research Station, Da’an, Jilin, China; 4 Center for Eco-Environment Restoration Engineering of Hainan Province, School of Ecology, Hainan University, Haikou, China; 5 China Railway 14th Bureau Group Northwest Engineering Co., Ltd., Xi’an, China; 6 National Key Laboratory of Black Land Protection and Utilization, Northeast Institute of Geography and Agroecology, Chinese Academy of Sciences, Changchun, China; 7 National Saline-Alkali Land Comprehensive Utilization Technology Innovation Center, Northeast Saline-Alkali Land Sub-Center, Da’an, Jilin, China

**Keywords:** growth, *Oryza sativa* L., salinization, sodification, soil heterogeneity, yield

## Abstract

Soil salinity, sodicity, and alkalinity are frequently intensified by field-scale heterogeneity characterized by uneven spatial distributions of salts, moisture, and nutrients. In rice systems under sodic and saline-sodic soil conditions, such heterogeneity leads to uneven crop stands, variable plant responses, and challenges in applying uniform management practices. Worse, such fields receive different amounts of amendments, while similar field management practices are frequently supplemented, reducing the efficiency of amelioration. Meanwhile, field operations such as land leveling and ploughing further redistribute salts, probably creating new heterogeneity patterns. Currently, traditional methods fail to address these complexities, resulting in inconsistent growth, inefficient resource use, and variable yields. Despite these challenges, no systematic review has addressed them. This review fills the gap by examining how spatial variability in physico-chemical and biological factors affects rice performance at critical growth stages. It also evaluates integrated strategies, including organic/inorganic amendments, irrigation and drainage, and rice varieties and their cultivation to improve rice productivity under these conditions. Our review suggests focusing on the interaction between soil heterogeneity and plant growth, and on integrating plant and soil-based management strategies with site-specific technologies, with particular focus on the critical growth stages of rice, where targeted interventions can significantly and effectively enhance rice performance in heterogeneous sodic/saline-sodic soils.

## Graphical Abstract

**Figure:**
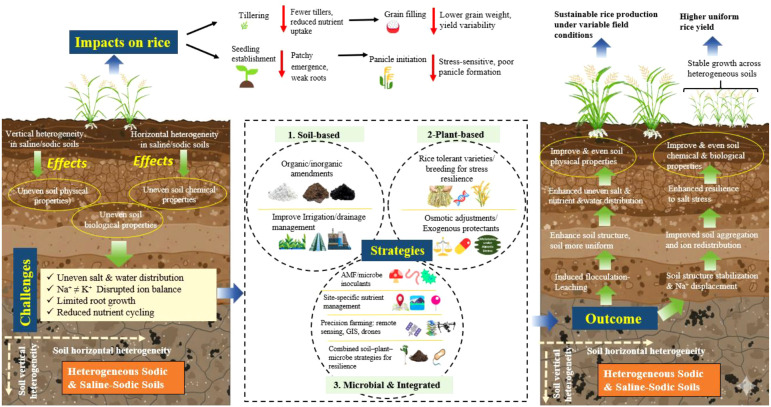

## 1 Introduction

Soil salinization, sodification, and alkalinization are major abiotic stresses that severely deteriorate soil health, restrict nutrient availability, and reduce crop productivity (Huang et al., 2017; Tiggeloven et al., 2020). Salt-affected soils, including saline, sodic, and saline-sodic types, are globally widespread, impacting over one billion hectares across more than 100 countries (Qadir et al., 2007; Hopmans et al., 2021; Shah et al., 2025). According to the FAO (2020), approximately 3% of the global topsoil (0–30 cm) and over 6% of the subsoil (30–100 cm) are salt-affected. These soils occur mainly in arid, semi-arid, and coastal regions but can also develop in poorly drained irrigated lands and areas with saline groundwater tables (Ali et al., 2014; Wang et al., 2024). The problem is expected to intensify with global climate change, leading to increased water scarcity, sea-level rise, and poor soil management practices (Ivushkin et al., 2019; Mukhopadhyay et al., 2023). Notably, around 60% of salt-affected soils are classified as sodic or saline-sodic, and these soil types are widely distributed in agricultural regions (Qadir et al., 2007; Rengasamy, 2010; FAO et al, 2020; Hossain et al., 2020; Wang et al., 2021). The largest extents of such alkaline, sodic, sodic-saline soils occur in countries such as India, China, the USA, Pakistan, and Australia (Alharby et al., 2018). Reportedly, India has over 3 million hectares of sodic soils, mainly in the Indo-Gangetic Plains, while China reports over 7 million hectares, particularly in the Songnen Plain (Abbas et al., 2021). In Australia, about 33% of agricultural land is affected by sodicity, highlighting the severity of the problem (Chi et al., 2012; Chhabra and Chhabra, 2021; Huang et al., 2022; Rezapour et al., 2022). Other affected regions include Argentina (34% of irrigated land), South Africa (18%), and Egypt (33%) (Singh et al., 2013; Wu et al., 2024). Worse, China incurs annual losses of about USD 410 million while India loses nearly USD 3 billion due to such salinity-affected soils, including sodic/saline-sodic ones (Hussain et al., 2017).

At the soil level, sodic soils have ESP above 15%, low EC, and a high pH usually exceeding 8.5 (Chi et al., 2012; Huang et al., 2022). Saline-sodic soils combine the properties of both, with EC values above 4 dS m^-1^ and ESP above 15% (Hafez et al., 2021; Liu et al., 2024). **Table 1** shows the classification of soil affected by salinity and sodicity based on EC, SAR, and pH. Sodic and saline-sodic soils are primarily distinguished by their elevated sodium levels, which alter soil structure and disrupt its chemical properties (Singh et al., 2013; Huang et al., 2022). Excess sodium on the soil exchange complex leads to dispersed soil structure, reduced aggregate stability, and decreased water infiltration and hydraulic conductivity (Brady and Weil, 2008; Choudhary and Kharche, 2018; Verma et al., 2024). Then, these conditions cause surface crusting, poor aeration, and root penetration problems (Huang et al., 2017). Noteworthy, high pH in sodic soils is mainly due to the presence of carbonates (CO_3_²^-^) and bicarbonates (HCO_3_^-^), which also decrease the solubility of essential nutrients such as phosphorus (P), zinc (Zn), iron (Fe), and manganese (Mn) (Dutta et al., 2021). In saline-sodic soils, the simultaneous presence of high salt concentrations and exchangeable sodium creates complex interactions that impair root function, increase osmotic stress, and disrupt ion homeostasis (Osman, 2018). Management strategies should be tailored accordingly; saline soils often respond well to leaching, sodic soils require sodium replacement with calcium source, and saline-sodic soils require integrated approaches addressing both salinity and sodicity. Understanding this variability is crucial for both elucidating plant-soil interactions under stress and developing site-specific and integrated management strategies. In fact, the spatial heterogeneity of soil properties, including pH, EC, and SAR, in a horizontal and vertical way at field, farm, and regional tempo-spatial scales, is being focused on (Booltink et al., 2001; Wang et al., 2017; Alharby et al., 2018; Khan et al., 2023). In essence, spatial variability in salinity, sodicity, moisture, and nutrient levels creates an uneven environment that complicates agricultural management (Chuamnakthong et al., 2019; Suska-Malawska et al., 2022). For example, such heterogeneity at the field scale leads to non-uniform crop growth, inconsistent response to fertilizers and soil amendments, and inefficient use of water resources (Haj-Amor et al., 2022). However, several practical challenges remain unresolved. (1) Fields with different sodic, saline-sodic levels are treated differently in terms of amendment amounts and amelioration strategies, but still receive similar nutrient supplements, e.g., fertilizers, water management practices, e.g., irrigation, rice cultivars, and plant densities inputs (Choudhary and Mavi, 2019). (2) During field operation, such as ploughing and land leveling, high stress-affected patches mix with less stress-affected zones, and redistribution of salts creates new soil heterogeneity patterns that complicate the identification, targeting, and management of specific heterogeneous zones (Ivushkin et al., 2019). (3) Although blocks within a field are designed to be of a uniform size, no standard method or practice exists to manage spatial heterogeneity within them, limiting the effectiveness of treatments at a fine scale (Osman, 2018). Consequently, traditional uniform management approaches fail to meet the practical requirements of heterogeneous sodic and saline-sodic fields due to these complexities. Although no universally accepted standard method exists, effective management generally involves characterizing spatial and vertical variability identifying critical stress zones and sensitive growth stages, and integrating appropriate soil, water, and crop management strategies, which is the focus of this review.

**Table 1:** Classification and distinctive properties of saline, sodic, and saline-sodic soils, modified from Rengasamy (2010).

Soil class	Soil subclass	EC (dS m^−1^)	pH	SAR/ESP	Key chemical properties	Key physical /structural properties	Field description
Normal soil	–	< 4	6-8	< 6	Balanced ion composition	Stable structure; good permeability	There is no visible salt accumulation; uniform crop growth pattern
Saline soil	Acidic	> 4	< 6	< 6	High soluble salts; micronutrients e.g., Fe, Al, toxicity	Osmotic stress	Salt patches/crust; growth restrictions
	Neutral	> 4	6-8	< 6	Soluble salt dominates; possible ion toxicity at high EC	Osmotic stress	Variable salt patches; restricted crop growth
	Alkaline	> 4	> 8	< 6	CO_3_^2-^ HCO_3_^−^ toxicity; Fe, Al, Mn, deficiencies/toxicities at pH> 9	Osmotic stress	White crusts on soil surface; poor plant growth
Sodic soils	Acidic	< 4	< 6	> 6	High exchangeable micronutrients e.g., Fe, Al toxicity	Soil structure instability; waterlogging risks	Patchy growths; restricted drainage
	Neutral	< 4	6-8	> 6	Excess Na^+^; anion toxicity e.g., Cl^-^, SO_4_^2−^	Clay dispersion; low infiltrations	Waterlogging; poor root penetration
	Alkaline	< 4	> 8	> 6	Na^+^, CO_3_^2−^, HCO_3_^−^ toxicity; micronutrient imbalance	Severe dispersion; poor soil stability	Soil surface crusting; poor seed germination
Saline-sodic soil	Acidic	> 4	< 6	> 6	High salts and Na^+^ contents; toxicity of micronutrients	Osmotic stress plus Na^+^ toxicity	Visible salts; poor growth
	Neutral	> 4	6-8	> 6	High soluble salts plus Na^+^; ionic toxicities	Osmotic stress	Salt patches; reduced crop growth
	Alkaline	> 4	> 8	> 6	High soluble salts plus Na^+^; CO_3_^2−^, HCO_3_^−^ toxicity; micronutrients issues	Severe osmotic stress plus; structural instability	Patchy growth; severe yield loss

Rice is the most important food crop in the developing world, serving as a staple cereal for more than 50% of the global population (Alkahtani et al., 2020). While rice is often cultivated in sodic and saline-sodic areas due to its superior tolerance compared to other common crops, it remains generally sensitive to high levels of soil salinity and alkalinity, which significantly restrict yield (Scherer et al., 1996; Rengasamy, 2010; Alonge et al., 2019). Specifically, although rice is comparatively more tolerant to sodicity than glycophytes, its productivity is severely influenced under extreme sodic conditions (Singh et al., 2016; Guo et al., 2023). Furthermore, rice is more sensitive during the seedling and reproductive stages when exposed to saline and sodic soil conditions (Kakar et al., 2019; Liu et al., 2023). Despite its sensitivity, rice is often considered a suitable crop for desalinization efforts due to its ability to thrive in flooded conditions, which facilitate the leaching of salts from the root zone, enhance nutrient mobilization, and stimulate microbial activity in the rhizosphere through its root system (Qadir et al., 2007; Daliakopoulos et al., 2016; Xu et al., 2020). Moreover, according to Nassar et al. (2020), rice’s shallow rooting depth reduces its exposure to elevated ES in deeper soil layers. However, the uneven distribution of stresses across the field often negates these benefits, leading to poor seedling establishment, reduced tillering, stunted growth, and low grain yield, especially during critical stages including germination, flowering, and grain filling (Zhao et al., 2020). Rice yields in sodic, saline-sodic soils remain low and inconsistent, ranging from 0.7 to 4.9 t ha^-1^, which is well below the global average irrigated yield of 5.4 t ha^-1^ (Wang et al., 2014; Zhao et al., 2020), although various reclamation practices including chemical amendments, manuring, improved water management, and sand application, have been applied (Wijitkosum et al., 2020). This indicates that conventional approaches often fail to fully address the underlying soil constraints in both horizontal and vertical ways. With the global population projected to exceed 9 billion by 2050, reclaiming and managing sodic, saline-sodic soils is not only a scientific challenge but also a socio-economic imperative (Choudhary and Mavi, 2019). Under such background, it is essential to summarize the characteristics of soil heterogeneity, its effects, and management strategies to reduce soil constraints and enhance rice yield and soil fertility. To our knowledge, no previous review has comprehensively examined rice growth and yield formation in heterogeneous sodic and saline-sodic soils. Based on this context, this review aims to highlight the heterogeneity of key soil constraints, their impacts on rice productivity, and to evaluate effective management strategies, while proposing adaptive management strategies that account for soil heterogeneity to support sustainable rice cultivation in degraded soils.

## 2 Soil heterogeneity in saline-sodic soil conditions

### 2.1 Horizontal soil heterogeneity

#### 2.1.1 Formation of horizontal soil heterogeneity

**Figure 1:**
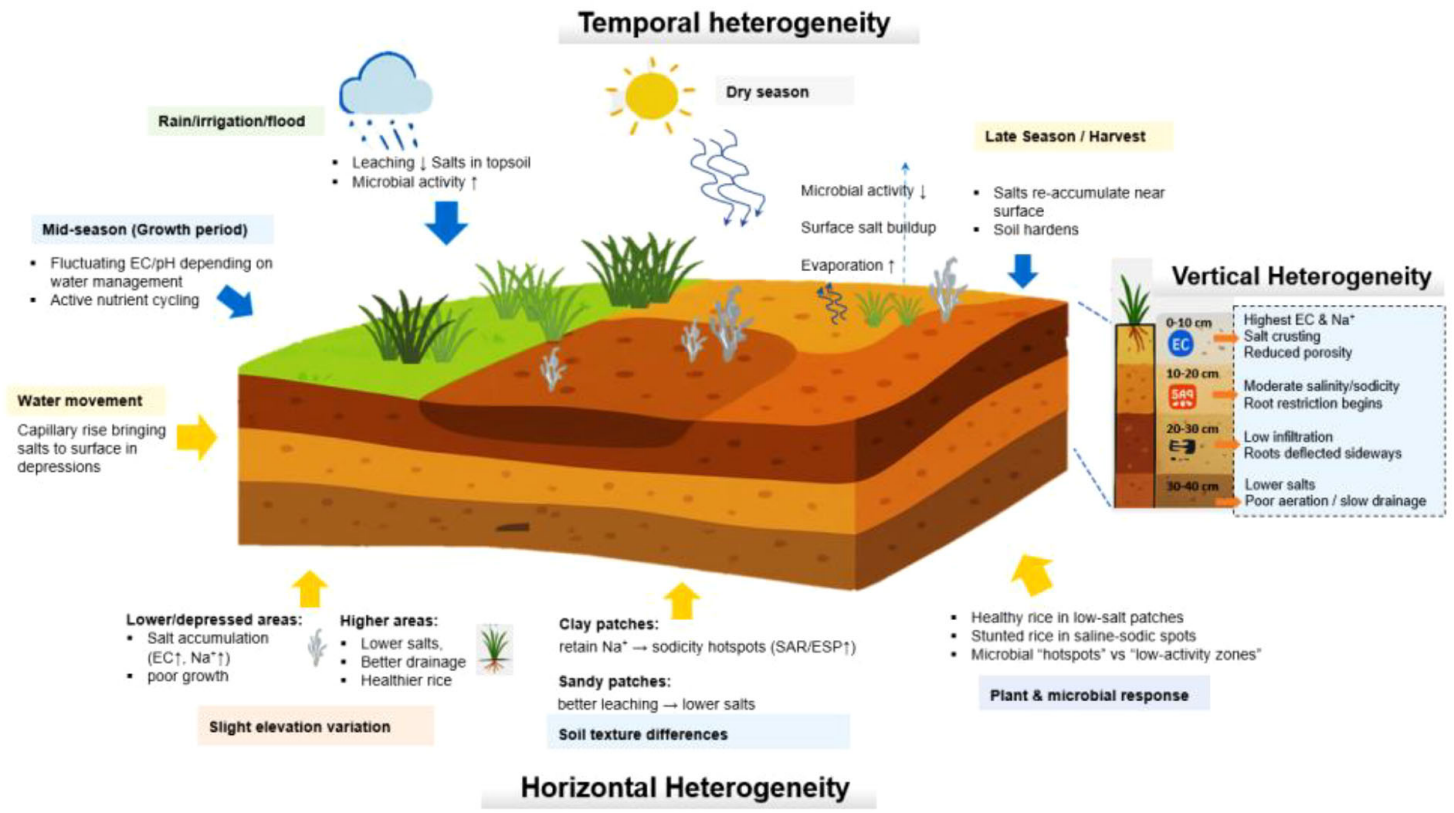
The figure illustrates the combined influence of horizontal, vertical, and temporal heterogeneity on soil properties, plant response, and microbial activity. Horizontal variability arises from slight elevation differences, soil texture variations, and water redistribution, leading to contrasting zones such as salt-affected depressions and better-drained elevated patches. Vertical heterogeneity reflects layered differences in salinity, sodicity, soil structure, and root responses from topsoil to deep horizons. Temporal variation is driven by seasonal cycles of rainfall, flooding, irrigation, drying, and evaporation, which influence salt leaching, accumulation, nutrient cycling, and microbial hotspots. These factors create highly patchy soil environments that regulate plant growth, salinity dynamics, and ecosystem functioning.

Soil heterogeneity refers to the uneven distribution of chemical, physical, and biological properties across space and time (Rezapour et al., 2022; Juleel et al., 2023). This variability is a common feature of natural soils but is especially pronounced in sodic and saline-sodic soils (Ondrasek and Rengel, 2021). The most visible feature is the irregular spatial distribution of salts, sodium, and alkalinity, forming patchy zones with contrasting soil conditions (Booltink et al., 2001). These patches appear as differences in soil color, surface crusting, or moisture content (Bazihizina et al., 2012). Horizontal variability develops through natural processes such as topographic gradients, hydrological flows, sediment deposition, and human agricultural practices (**Figure 1**) (Alharby et al., 2018; Delbari et al., 2019). Topography strongly shapes horizontal heterogeneity, as even slight elevation differences redistribute water, salts, and sediments, forming microzones with distinct properties (Kumawat et al., 2022). Nguyen et al. (2014) reported that higher-elevation areas receive more freshwater, while lower-elevation zones accumulate saline irrigation or natural seepage, creating variability within the same field. Similarly, in inland regions, shallow depressions often act as sink for salts transported through capillary rise or irrigation return flows while elevated positions remain comparatively less affected, resulting in a mosaic of saline and less saline patches (Dinneny, 2019). Recent field observations from irrigated marsh landscapes in Spain also demonstrated that irrigation return flows and variable irrigation water quality can produce marked salinity heterogeneity at the field scale, even under uniform cropping, highlighting the role of irrigation management in creating spatially variable salt patterns across agricultural fields (Egea et al., 2026).

Variability in soil texture also contributes to horizontal heterogeneity, as clay-rich zones retain sodium and drain poorly, while sandy soils promote faster salt leaching (Aldabaa et al., 2015). Such patterns reflect long-term geomorphic processes such as flooding, erosion, and sediment deposition (Zhou et al., 2024), indicating that present-day heterogeneity frequently reflects legacy soil-forming processes that predate current management. Human activities, including uneven tillage, ploughing, land levelling, and irregular irrigation combined with poor drainage or canal leakage, further intensify this variability by redistributing salts and organic matter across fields (Wang et al., 2023). The repeated use of brackish groundwater or low-quality irrigation water in certain parts of a field can exacerbate salt build-up locally, while zones closer to freshwater inlets may maintain comparatively lower salinity, reinforcing patchiness in salinity distributions and contributing to field-scale heterogeneity (Alharby et al., 2018). Fertilizer and amendments are also often applied unevenly, especially in smallholder systems, which further accentuates the patchy distribution of soil fertility and sodicity (Khan et al., 2023). In addition, historical land use practices such as grazing, manuring, crop rotations, and puddling leave persistent soil features including hardpan layers at different depths, which influence surface and subsurface salt accumulation patterns (Booltink et al., 2001). Long-term crop rotations that integrate rice with perennial pastures and livestock grazing further shape spatial soil properties by diversifying root architectures and organic matter inputs, which can buffer salt redistribution over time and promote more stable physical conditions compared with continuous rice monoculture (Lattimore, 1994; Vecchio et al., 2018). Biological heterogeneity also emerges as microbial communities, organic matter turnover, and root activity differ across micro zones due to variations in moisture, oxygen availability, and salt levels (Dinneny, 2019). For instance, zones with higher organic residue inputs often promote greater microbial activity and organic acid production, which locally modifies soil pH and sodium mobility (Rezapour et al., 2022). Recent advances in digital soil mapping, remote sensing, and geostatistical modelling now allow this horizontal heterogeneity to be quantified and visualized with increasing accuracy, proving valuable tools for understanding its formation and dynamics at the field scale and for designing spatially targeted management strategies (Gupta et al., 2020, Gupta et al., 2022).

#### 2.1.2 Mechanistic implications for rice growth

**Figure 2:**
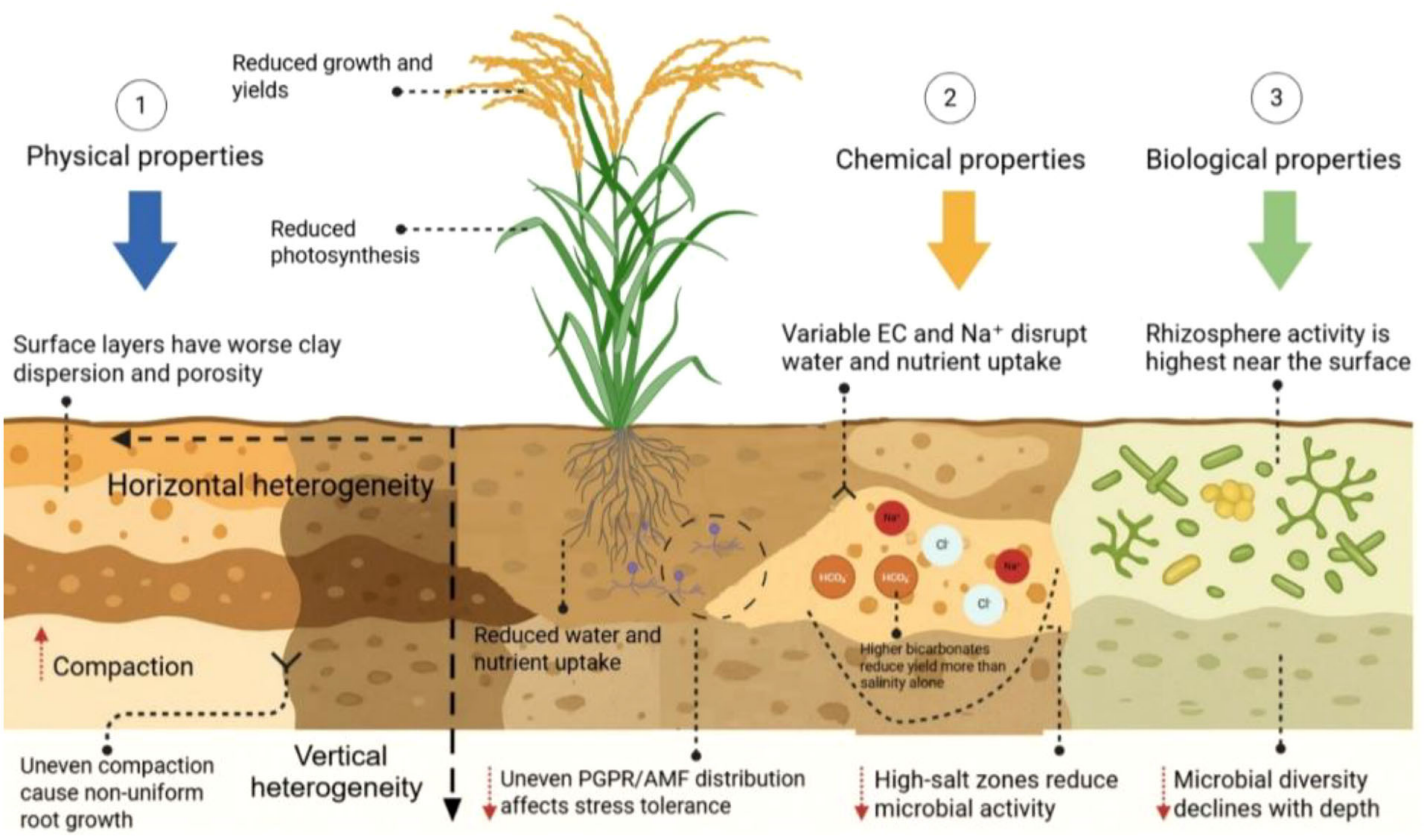
Illustrative diagram showing the effects of soil heterogeneity on physical, chemical, and biological properties in sodic and saline-sodic soils. Soil heterogeneity is represented by variations in color and texture: darker brown shades indicate high levels of compaction and clay dispersion (physical constraints); lighter/yellowish patches represent concentrated zones of salts (Na^+^) and bicarbonates (HCO_3_^-^) (chemical constraints); and green-tinted zones highlights areas of higher rhizosphere microbial activity. Variation in these properties restrict root development, water and nutrient uptake, and microbial activity. These stressors collectively reduce photosynthesis and stress tolerance, leading to lower rice productivity.

Horizontal heterogeneity in saline-sodic soils strongly influences rice growth through the uneven distribution of salts, nutrients, and moisture across the field (Bazihizina et al., 2012; Liu et al., 2024; Yazhini et al., 2024). Chemical soil properties such as pH, EC, SAR, ESP, nutrient levels, and toxic ions (Na^+^, Cl^-^, HCO_3_^-^) can change sharply over short distances (Mandal et al., 2008; Xu et al., 2020; Liu et al., 2024). These chemical imbalances disrupt nutrient availability and create localized zones of stress, resulting in patchy rice establishment and growth (**Figure 2**). In particular, areas enriched with Na^+^, HCO_3_^-^ promote clay dispersion, poor aggregate stability, and reduced infiltration, which collectively create drought-like conditions in certain patches despite sufficient irrigation (Hafez et al., 2021). Such fine-scale chemical variability not only limits uniform rice establishment but also complicates field-level management practices.

Similarly, physical heterogeneity further modifies rice growth by altering soil bulk density, porosity, and texture, which regulate water movement, aeration, and root penetration (Mandal et al., 2008). The areas with clay-rich or compacted layers restrict infiltration and promote localized salt build-up, while sandy or well-structured areas enable leaching and maintain lower salinity and sodicity (Riaz et al., 2019). As a result, dynamic microenvironments develop within the root zone, where moisture, salinity, and oxygen levels fluctuate rapidly following irrigation or rainfall events (Preece and Peñuelas, 2020; Verma et al., 2024). Seasonal changes in bulk density and porosity, including surface crusting during dry periods and improved infiltration after wetting, further modify nutrient diffusion and availability to roots (Chuamnakthong et al., 2019). Also, variability in infiltration and water retention causes uneven salt leaching, intensifying spatial heterogeneity in salinity and sodicity (Singh et al., 2016; Hu et al., 2017). Consequently, patches with high EC and poor aeration restrict root density and nutrient uptake, while adjacent low-sodicity zones provide more favourable growth conditions (Li et al., 2017; Delgoda et al., 2023; Zhou et al., 2024).

Soil biological properties exhibit similar variability, as microbial diversity, enzymatic activity, and community composition respond to micro-scale shifts in salinity, sodicity, and moisture (Coonan et al., 2020). These uneven biological properties have strongly implications for rice growth because microbial processes regulate nutrient cycling, root colonization, and stress mitigation (**Figure 2**) (Yuan et al., 2007). High saline or compacted sodic patches often host reduced microbial biomass and enzymatic activity, forming “microbial deserts” that limit nutrient transformation and availability (Wijitkosum, 2020). In contrast, less-stressed areas support greater microbial richness, including beneficial groups such as arbuscular mycorrhizal fungi (AMF) and plant growth-promoting rhizobacteria (PGPR) (Sardinha et al., 2003). This patchy microbial distribution reinforces variability in nutrient supply, water use efficiency, and root development (Qin et al., 2016). Field studies show that bacterial and fungal communities can shift markedly over short distances (10–50 m) in sodic paddy soils, with some patches dominated by halotolerant taxa and others by plant growth-promoting microbes, leading to yield differences of up to 25% within the same field (Alharby et al., 2018; Feng et al., 2019; Khan et al., 2023; Wang et al., 2023). Such heterogeneity complicates fertilizers and water management because uniform applications fail to meet localized crop demands (Booltink et al., 2001). Currently, research on field-scale horizontal variability in rice paddies remains limited, with many studies still relying on spatially averaged measurements that obscure the true impacts of salinity and sodicity heterogeneity on rice performance (Dinneny, 2019).

### 2.2 Vertical soil heterogeneity

#### 2.2.1 Formation of vertical soil heterogeneity

Vertical soil variability refers to depth-dependent differences in physical, chemical, and biological properties along the soil profile (Corwin et al., 2003; Ashoka et al., 2023). It is most evident in gradients of salinity, sodicity, and alkalinity, which develop through the interaction of natural processes and land management practices (Bazihizina et al., 2012; Suska-Malawska et al., 2022). These gradients arise as salts move upward through evapotranspiration and capillary rise or downward through rainfall and irrigation-induced leaching, with soil properties regulate their redistribution and retention (Wang et al., 2017; Kashenge-Killenga et al., 2016). Seasonal fluctuations in water table and irrigation patterns further reinforce these gradients by altering the balance of salt and moisture within the profile (Qin et al., 2016). Multiple studies highlighted that soil salinity and sodicity levels can change markedly in response to rainfall variability, irrigation practices, and amendment applications, with climate factors often emerging as dominant controls on these shifts (Bazihizina et al., 2012; Li et al., 2022a). Furthermore, high salinity and sodicity are usually concentrated in the top 0-10cm layer due to evaporation, capillary rise, and insufficient surface water ponding, which lead to surface crust formation and surface sealing (Shahid et al., 2018; Liao et al., 2020). The 10–20 cm layer often retains higher levels of sodicity, while deeper layers (below 20 cm) generally exhibit lower salt concentrations, although compaction, poor drainage, and past waterlogging can still generate localized stress zones (Chi et al., 2012). The vertical distribution of salts is strongly influenced by soil texture, clay content, and cation exchange capacity, which regulate the transport and retention of soluble salts along the profile (Schubert and Qadir, 2024). In parallel, physical heterogeneity such as soil structure, bulk density, and porosity varies with depth and further shapes vertical heterogeneity (Yang et al., 2011). Dense or compacted subsoils limit water percolation and promote salt accumulation in upper horizons, and strengthen chemical gradients (Schubert and Qadir, 2024). Soil texture variations, such as clay-rich sublayers, further intensify vertical heterogeneity by trapping salts and modifying moisture retention (Wang et al., 2014; Osman, 2018). Furthermore, variation in root density and root exudation contributes to biological heterogeneity by altering microbial habitats and nutrient availability at different depths (Wang et al., 2017, Wang et al., 2024). The combined effects of chemical, physical, and biological stratification create a vertically layered environment that strongly influences root distribution, water uptake, and plant exposure to stress (Zhou et al., 2024). Despite its importance, most studies focus primarily on the upper 0–20 cm of the soil layer, with limited attention to deeper horizons (30-100), where critical constraints and processes persist (Liao et al., 2020). These subsoil layers can store significant amounts of salts, nutrients, and water, influencing long-term soil functioning, crop performance, and hydrological connectivity (Huang et al., 2022). Consequently, a comprehensive full-profile characterization is essential to understand salt accumulation patterns, drainage limitations, and nutrient cycling in saline-sodic systems (Wang et al., 2014).

#### 2.2.2 Mechanistic implications for rice root and yield processes

Vertical heterogeneity in saline, sodic, and saline-sodic soils strongly affect soil functions and rice performance by creating depth-wise variation in chemical, physical, and biological properties (Qadir et al., 2007; Ahmadi and Souri, 2020). Chemical gradients are especially evident for soil pH, salinity, and sodicity indicators, nutrient levels, and harmful ion concentrations. Salts and alkaline ions commonly accumulate in the upper 0–20 cm due to limited leaching, capillary rise, and inadequate surface ponding, which elevates soil pH and reduces nutrient availability (Qadir et al., 2006; Hafez et al., 2021). These surface conditions restrict early root establishment, nutrient uptake, and photosynthetic efficiency, resulting in reduced tillering and biomass accumulation in rice (**Figure 2**) (Mandal et al., 2008; Roy et al., 2014). Elevated Na^+^ and carbonate/bicarbonate levels further disrupt ion homeostasis in plant tissue, affecting nutrient absorption and metabolism processes (Roy et al., 2014). In contrast, deeper soil horizons generally show lower salinity but are constrained by physical barriers such as compaction, poor drainage, and dense subsoil layers (Liu et al., 2023). These limitations restrict root penetration, water movement, and nutrient exploration, which collectively reduce rice growth potential and field-level productivity (Daliakopoulos et al., 2016). Vertical gradients in EC, pH, total carbon (TC), nitrogen content, C/N ratio, and exchangeable Ca^2+^, Mg^2+^, and Na^+^ have been reported even in short depth intervals, whereas available phosphorus and exchangeable potassium show high variability, even in relatively flat fields (Yana et al., 2000; Delgoda et al., 2023).

Furthermore, in sodic fields, the upper soil layer (0–20 cm) often exhibits severe sodicity and high bulk density, forming surface crusts that reduce porosity and infiltration (Jackson and Caldwell, 1996; Osman, 2018). These conditions exacerbate surface runoff, erosion, and localized waterlogging (Cemek et al., 2007). Beneath the compacted layer, poorly drained subsoil further restricts oxygen availability, although deeper layers may contain lower salt concentrations; they remain underutilized due to the barrier effect imposed by degraded surface layers (Speirs, 2010; Wang et al., 2017; Choudhary and Mavi, 2019). Moreover, the surface layers support higher microbial diversity, biomass carbon, and enzymatic activity, while subsoil layers accumulate salts, and organic matter depletion leads to reduced microbial abundance, altered community compositions, and suppressed enzyme activity (Yao et al., 2021). Kumawat et al. (2022) reported that microbial diversity and activity decline with depth due to lower organic matter availability, reduced oxygen diffusion, and greater salt accumulation. Consequently, surface layers function as hotspots of rhizosphere interactions, while subsurface horizons act as biological “deserts” characterized by microbial dormancy or mortality (Kumar et al., 2022). Li et al. (2020) highlighted that microbial biomass carbon and nitrogen declined by more than 40% in subsoil layers compared to surface layers of saline-sodic soils, while urease and phosphatase activities were reduced by nearly half. This vertical imbalance in microbial activity influences rhizosphere processes such as exudate decomposition, nitrogen transformation, and pathogen suppression, indirectly affecting rice growth and yield formation (Roy et al., 2014). Overall, the interaction among surface salt accumulation, soil compaction, and subsoil biological limitations generates complex challenges on root distribution, water uptake, and nutrient acquisition, posing major challenges for effective management of these systems. From a management perspective, horizontal and vertical soil heterogeneity require different intervention strategies. Horizontal heterogeneity is best addressed through spatially targeted, field-scale practices, such as localized amendment application, variable water management, and zoning of saline or sodic patches. In contrast, vertical heterogeneity requires depth-oriented interventions, including subsoil amelioration, improved drainage, deep leaching, and practices that enhance root penetration across restrictive layers. While horizontal heterogeneity mainly drives within-field variability in crop performance, vertical heterogeneity often limits the overall effectiveness of surface-applied treatments by restricting root access to deeper soil resources.Fageria, 20072.3 Temporal soil heterogeneity.

### 2.3 Temporal soil heterogeneity

#### 2.3.1 Drivers and dynamics

Temporal soil heterogeneity describes changes in soil properties over time that influence the distribution of salt and nutrients (Oliveira and Scheffers, 2019). These dynamics arise from the interaction among climatic factors, irrigation schedules, water management, crop cycles, and microbial activity (**Figure 1**) (Ali et al., 2014). For instance, rainfall events can temporarily dilute surface salts, whereas periods of high evaporation concentrate salts at the soil surface and in shallow horizons (Fageria et al., 2006; Fageria, 2007). Seasonal patterns such as during monsoon or wet seasons, surface leaching reduces salinity in the upper soil layers, while dry seasons promote salt accumulation through evapotranspiration and capillary rise (Wijayanto et al., 2023). Similarly, floods and droughts add further fluctuations, interacting with soil texture and microtopography to create both predictable seasonal trends and stochastic variability (Roy et al., 2014). Other, irrigation timing, method, and quality of water further shape these dynamics by controlling soil moisture and salt redistribution (Chhabra and Chhabra, 2021). For example, sodium-rich canal or groundwater irrigation can temporarily elevate surface sodicity, whereas subsequent freshwater irrigation may partially reverse these effects, resulting in dynamic chemical gradients (Singh et al., 2016). Repeated wetting and drying cycles alter soil physical properties through clay swelling and shrinkage, crack formation, and changes in porosity, affecting water and solute movement over time (Anil et al., 2005). These changes are especially pronounced in paddy soils due to the alternating flooded and drained conditions, which modify redox potential, nutrient availability, and seasonal salt distribution (Qadir et al., 2007; Morales et al., 2011; Chhabra and Chhabra, 2021). Hydrological processes such as capillary rise transport salts upward during dry periods, while rainfall-driven subsurface flow redistributes salts both horizontally and vertically (Bazihizina et al., 2012; Wijayanto et al., 2023). Biologically, organic inputs such as crop residues or manure decompose at different rates, causing fluctuations in nutrient availability and pH (Siddique et al., 2020). Nguyen et al. (2014) studied that microbial and enzymatic activities respond dynamically to changes in moisture, temperature, and salinity, altering nitrogen mineralization, phosphorus availability, and organic matter turnover throughout the growing season. These interactions create feedback loops in which biological processes both respond to and modify soil chemical and physical conditions (Li et al., 2022a). Understanding these temporal patterns is crucial for predicting soil behavior and designing adaptive management strategies in saline-sodic affected rice systems.

#### 2.3.2 Consequences of temporal soil heterogeneity

Temporal heterogeneity in sodic and saline-sodic soils generates shifting chemical, physical, and biological conditions that strongly influence rice establishment, nutrient uptake, and yield stability (Qadir et al., 2007; Ahmadi and Souri, 2020; Chhabra and Chhabra, 2021). Chemical properties, e.g., pH, EC, SAR, ESP, and nutrient availability, change over time, creating periods of intensified stress during sensitive rice growth stages (Qadir et al., 2006; Hafez et al., 2021). For example, EC often increased from the vegetative to ripening stage due to evapotranspiration, while pH fluctuates through water management and nutrient transformations, altering ion solubility and nutrient availability (Qadir et al., 2007; Barrera et al., 2020). Seasonal patterns have revealed rising EC in low-lying fields during January to September, while higher-elevation areas maintain relatively stable pH levels, highlighting stage-specific salt stress (Nguyen et al., 2014). Furthermore, temporal variation in micronutrient availability, such as Fe, Mn, and Zn across growth stages (pre-sowing, bunch formation, and flowering), further contributes to stage-specific nutrient limitation (Morales et al., 2011). Also, long-term temporal trend indicates gradual soil acidification under intensive rice systems, reflecting cumulative management effects (Bazihizina et al., 2012; Siddique et al., 2020; Huang et al., 2022).

Soil physical properties fluctuate in response to rainfall, irrigation frequency, and evapotranspiration, shaping root environment over time (Xu et al., 2020). Wet periods improve infiltration and reduce crusting, while dry periods increase compaction, lower porosity, and raise mechanical resistance (Grzesiak et al., 2002; Hafez et al., 2021). These shifts restrict root elongation, alter biomass allocation, and impair water and nutrient acquisition, contributing to yield losses (Grzesiak et al., 2002; Kumawat et al., 2022). Alternating waterlogging and drought can create paradoxical stress cycles, limiting effective root functioning and narrowing optimal management windows (Mukhopadhyay et al., 2023). This hydrological imbalance, combined with the sticky texture of wet sodic soils and extreme hardness when dry, limits suitable periods for tillage, planting, and irrigation (Roy et al., 2014; Hopmans et al., 2021). However, current studies have improved the measurement of individual soil physical changes and their impact on plant performance, yet significant gaps remain (Riaz et al., 2019). Many studies focus on single factors under short-term conditions without incorporating multi-scale spatial-temporal variability into predictive models (Guo et al., 2015).

Furthermore, microbial biomass, diversity, and enzymatic activity vary with rainfall, irrigation, and salt accumulation, influencing nutrient cycling and root interactions (Preece and Peñuelas, 2020). Qu et al. (2020) showed that dehydrogenase and β-glucosidase activities in saline sodic paddy soils peak after monsoon irrigation but drop by nearly 60% during dry-season salt buildup, influencing nitrogen and carbon availability across stages, while seasonal shifts in microbial community composition also alter root-microbe interactions. Similarly, microbial biomass carbon and enzyme activities such as dehydrogenase and phosphatase also fluctuate with crop growth stages, with peak activity during early vegetative growth when root exudation was high, and declines under late season saline-sodic stress (Deng et al., 2011; Singh et al., 2016; Otlewska et al., 2020). These seasonal shifts alter rhizosphere processes and amplify yield variability, particularly in rice systems reliant on shallow roots and microbial nutrient cycling (Kumawat et al., 2022). Besides, Kumar et al. (2022) highlighted that rice is especially sensitive to such biological heterogeneity due to their shallow root systems. Barrera et al. (2020) reported that zones of higher microbial activity coincided with improved aggregation and organic carbon retention, while adjacent barren patches remained dispersive and nutrient-poor. Similarly, Wijayanto et al. (2023) demonstrated that biologically active patches act as nutrient hotspots, whereas low activity zones restrict root growth, reinforcing feedback loops that intensify spatial and temporal yield variability (Wijayanto et al., 2023). Despite advances in measuring individual soil processes, significant gaps remain in integrating multi-scale spatial-temporal variability into predictive models and management frameworks (Chhabra and Chhabra, 2021). Addressing these gaps is essential for improving yield stability and sustainability in saline-sodic rice systems.

## 3 Rice growth responses to heterogenetic saline-sodic conditions

### 3.1 Seedling establishment

The seedling establishment stage is the most critical phase of rice growth in heterogeneous saline-sodic soils, where spatial and temporal variability strongly influence emergence and survival (Ali et al., 2014). In contrast to uniform stress, heterogeneous conditions create diverse microenvironments in which seeds and seedlings are exposed to variable and rapidly shifting stresses (Liu et al., 2010; Delgoda et al., 2023). Vertically, saline-sodic soils often consist of a relatively leached surface layer underlain by a compacted subsoil with high salinity and sodicity (Liu et al., 2010). Seedlings may establish successfully in the upper layer, but as roots elongate downward, they face a concentrated ionic barrier only a few centimeters below the surface (Hussain et al., 2017). This abrupt transition induces hydraulic shock, root cell apoptosis, and collapse of the developing root system, resulting in stunted growth or seedling mortality (Hopmans et al., 2021). Akbarimoghaddam et al. (2011) demonstrated that root elongation is severely restricted under these conditions, reducing the plant’s ability to explore the soil for water and nutrients, while excessive accumulation of toxic ions disrupts early photosynthetic activity and impairs enzymatic functions essential for seedling vigor. These effects are further intensified by the hard-setting nature of sodic subsoils, which impose both physical resistance and chemical toxicity, synergistically inhibiting root penetration (Shaddad et al., 2019).

Horizontally, soil heterogeneity manifests as a patchy mosaic of salinity across a field due to variability in irrigation efficiency, micro-topography, and soil texture (Akbarimoghaddam et al., 2011). Consequently, rice stands become highly irregular, with seedlings in favorable patches developing normally, while those in saline hotspots experience severe osmotic stress, tissue necrosis, and early death (Hopmans et al., 2021). It remains unclear whether reductions in rice growth and yield are primarily driven by the inherent heterogeneity of the soil or by the overall intensity of salinity in the field, highlighting the need for studies that disentangle these two factors. Field-scale investigations using electromagnetic induction (EMI) have quantified this patchiness, demonstrating that seedling mortality and vigor are directly correlated with the spatial scale and intensity of these saline-sodic patches. Establishment may fail completely in areas where surface EC exceeds genotype-specific tolerance thresholds (Srivastava et al., 2014), suggesting that the spatial pattern of salinity may be more damaging than uniform intensity. In uniformly saline fields, plants may adapt through systemic physiological adjustments; in contrast, in heterogenous field, abrupt transitions between low- and high-stress zones prevents stable adaptation, making heterogeneity a more significant driver of early-stage mortality than absolute salt concentration alone. In sodic patches, sodium-induced clay dispersion destroys soil aggregate stability, leading to surface crusting, reduced macroporosity, and impaired oxygen diffusion. These conditions create hypoxia that suppresses the high metabolic demands of germination and early growth, particularly when anaerobic stress coincides with ionic toxicity (Speirs, 2010).

The adaptive response of rice during this stage is mainly through root plasticity. During seedling establishment, roots redirect growth away from saline zones via halotropic responses and proliferation in favorable microsites, while deeper and more branched roots access fewer saline layers, offset vertical salinity gradients, and sustain growth under variable conditions (Ali et al., 2024). The capacity of a genotype to rapidly develop a deep radicle to bypass saline surface layers, or to promote lateral root growth in low-salinity patches, is therefore critical for successful seedling establishment (Rao et al., 2008). This foraging behavior is supported by cellular and biochemical adjustments (Hopmans et al., 2021). In stressful microsites, successful seedlings maintain cellular homeostasis by quickly accumulating compatible solutes such as proline and glycine betaine for osmotic balance, while precisely regulating cytosolic Na^+^/K^+^ ratio through transporters such as SOS1 and NHX transporters to mitigate ionic stress (Otlewska et al., 2020). Despite these advances, the genetic loci and molecular pathways that regulate root plasticity in response to subsurface salinity barriers or uneven salinity patches remain largely unknown (Ologundudu et al., 2014). Current research highlights a major limitation, as most insights into the physiological and genetic basis of salt tolerance during seedling establishment are derived from pot or hydroponic experiments under uniform stress. Such conditions fail to capture the complex three-dimensional interactions between soil and roots that determine seedling survival in heterogeneous field environments (Grzesiak et al., 2002). Furthermore, there is a notable shortage of phenotyping systems that can mimic controlled spatial salinity gradients to investigate root foraging behavior (Nassar et al., 2020). Future research should focus on field-based studies that capture the natural spatial variability in saline-sodic soils and integrate advanced geophysical soil sensing with high-resolution root phenotyping to better resolve plant-soil interactions during seedling establishment in heterogeneous soils.

### 3.2 Vegetative growth

**Figure 3:**
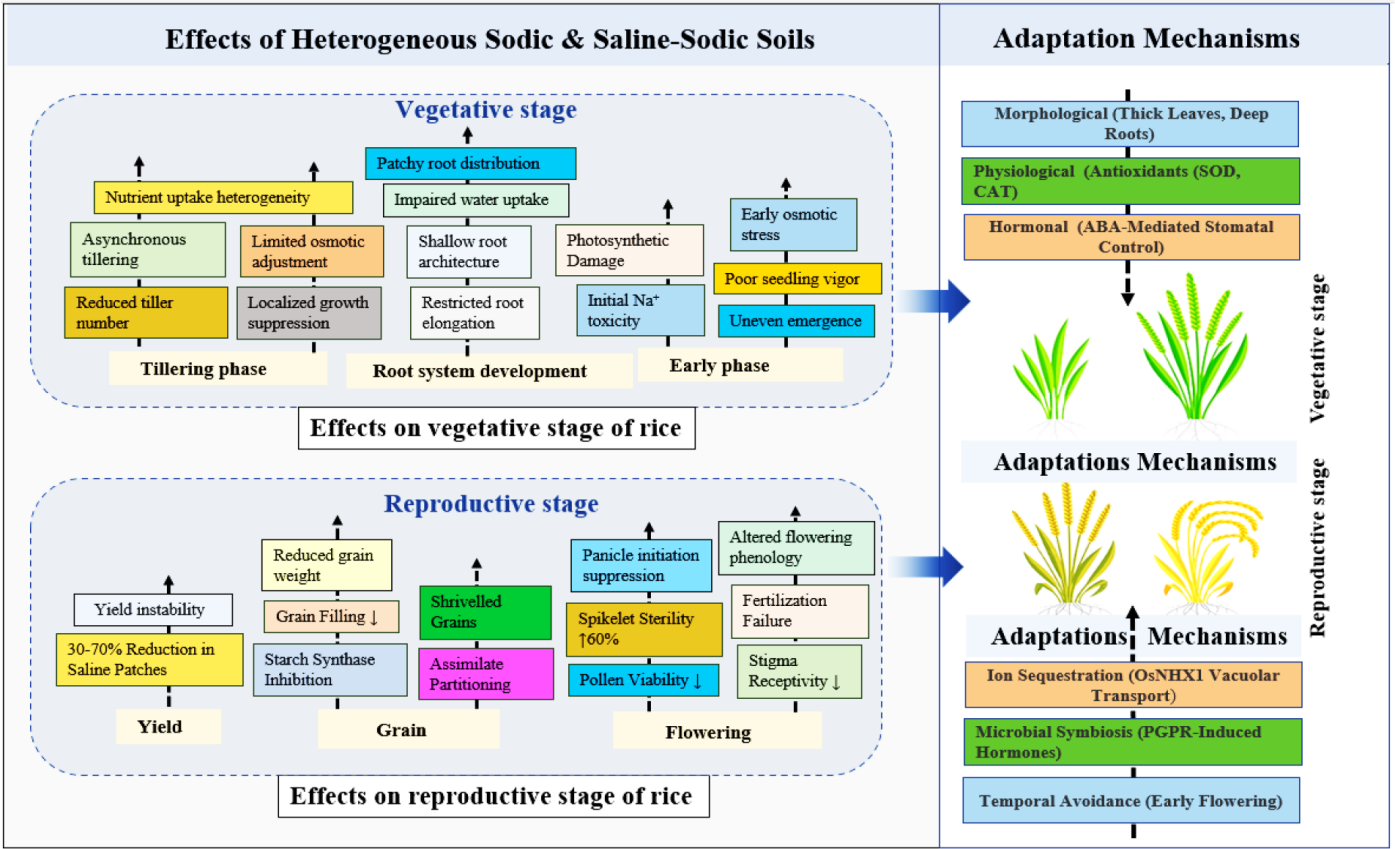
Schematic representation of the impacts of heterogenous sodic and saline-sodic conditions on the growth stages of rice and corresponding adaptation mechanisms. The figure summarizes the detrimental effects during the vegetative stage (e.g., impaired root development, photosynthetic damage) and the reproductive stage (e.g., spikelet sterility, reduced grain weight, and yield instability). The adaptive responses involve morphological, and physiological adjustments, hormonal regulation, ion sequestration, early flowering and microbial symbiosis.

Sodic and saline-sodic soils heterogeneity exhibit significant influence on rice vegetative growth due to their complex challenges, such as patchy distribution of salts, pH, and exchangeable sodium (Xu et al., 2020; Kumawat et al., 2022). These spatially variable constraints generate dynamic stress patterns that lead to uneven tiller development, leaf expansion, and non-uniform canopy structure (Kumawat et al., 2022). The effects of these constraints are shown in **Figure 3**. Within the root zone, nutrient availability is highly variable, as essential ions such as K^+^, Ca^2+^, and Mg^2+^ are displaced by Na^+^, disrupting osmotic regulation, turgor maintenance, and enzymatic activity, thereby impairing cell expansion and physiological functions (Chhabra and Chhabra, 2021). During the vegetative stage, rice exhibits strong plasticity to sodic and saline-sodic heterogeneity, driven by complex spatial and temporal soil patterns, which shape root architecture and canopy development and determine yield potential (Anil et al., 2005; Nassar et al., 2020). At the microscale, roots encounter sharp gradients in salinity and sodicity between soil aggregates, pores, and rhizosphere zones, where local variations in sodium accumulation and nutrient depletion strongly influence water potential and ion balance, forcing roots to selectively proliferate in relatively more favorable niches (Zhao et al., 2020). Studies using split-root designs have shown that rice roots can detect and avoid micro zones with EC exceeding 4 d/S m, while preferentially growing into nutrient-rich areas, demonstrating root plasticity as a key adaptive mechanism to heterogeneity (Zhao et al., 2020). Such root foraging strategies are crucial during vegetative growth because lateral root proliferation and aerenchyma formation enhance the capacity of rice to bypass toxic microsites while maximizing access to available water and nutrients (Kumar et al., 2007).

At the mesoscale, vertical heterogeneity in soil layers, such as a saline-sodic surface horizon over less-affected sublayers, shapes and root distribution and biomass allocation, resulting in an initial reduction of tiller number to prioritize deeper root exploration, a trade-off critical for later recovery and growth (Haj-Amor et al., 2022). A study using electrical resistivity tomography (ERT) has confirmed that tolerant rice varieties establish roots more rapidly and deeper in a less saline subsoil, effectively using it as a reservoir for water and nutrients (Arshad et al., 2017). This response to vertical heterogeneity is reflected in biomass allocation, where initial reduction of tiller number prioritizes deeper root exploration, a trade-off that is critical for subsequent recovery and growth (Bal et al., 2013). Rice often directs primary roots downward to escape surface sodicity while simultaneously adjusting tiller production to maintain shoot-root balance. This adaptive mechanism depends on the degree of heterogeneity, as extreme sodic crusting can block root penetration, whereas moderate patchiness allows selective rooting in the deeper, less saline layers (Choudhary and Mavi, 2019). At the macroscale, soil heterogeneity manifests across entire fields, where low-lying areas experience poor drainage and enhanced capillary rise, leading to pronounced contrasts in sodicity (Sheoran et al., 2021). Rice growing in favorable patches shows vigorous vegetative growth with higher tiller number and greater leaf area index, while plants in adjacent sodic patches remain stunted. This spatial variability produces heterogeneous canopies that alter light interception, photosynthetic efficiency, and competitive interaction among tillers (Chhabra et al., 2021).

Temporal heterogeneity during the vegetative stage further compounds these effects, as salinity and sodicity dynamics fluctuate with seasonal irrigation patterns, groundwater dynamics, and evapotranspiration (Hafez et al., 2021). Rice responds to these fluctuations by adjusting physiological traits such as stomatal conductance, osmolyte accumulation, and antioxidant defense, but the effectiveness of these responses depends strongly on stress timing (Kumar et al., 2007). Specifically, stress during early tillering can irreversibly suppress tiller initiation, whereas later exposure may reduce tiller survival while allowing some compensation through plasticity in leaf expansion and resource reallocation (Otlewska et al., 2020). The interaction of spatial and temporal heterogeneity produces a shifting stress landscape that require coordinated root foraging, shoot plasticity, and physiological resilience to sustain vegetative growth (Huang et al., 2022). Current studies have provided valuable insights into these adaptive mechanisms, but most studies are conducted under uniform or uncontrolled conditions, failing to capture the complexity of heterogeneous sodic and saline-sodic fields. Some genotypes succeed through deeper or more proliferated roots, while others rely on osmotic adjustment; however, the integration of these responses across spatial and temporal scales remains poorly understood. Significant gaps exist in linking microscale root-soil interactions to field-scale canopy dynamics, and temporal heterogeneity across successive vegetative phases is rarely considered. Future studies should adopt multi-scale and temporally explicit approaches to full capture rice adaptation in heterogeneous sodic and saline-sodic soils.

### 3.3 Reproductive development and yield formation

The reproductive stage is among the most sensitive phases of rice growth. In saline-sodic soils, rice performance during this stage is severely constrained, with soil heterogeneity further intensifying these effects (Khan and Abdullah, 2003). Such heterogeneity generates a mosaic of abiotic stresses that disrupt physiological processes that reduce flowering, fertilization, and grain filling, as a result reducing yield formation (Arshad et al., 2017; Irakoze et al., 2020; Razzaq et al., 2020). Vertically, restrictive saline-sodic layers are a major barrier to root proliferation that creates a hostile physical and chemical environment that impedes roots from accessing deep water and nutrients during critical key phenological stages such as flowering and grain filling (Khan and Abdullah, 2003; Chhabra and Chhabra, 2021). These constraints reduce assimilate supply to reductive organs and increase the risk of yield loss. Horizontally, patchy distribution of high ESP and Ece generates sharp spatial variability in plant-available water and osmotic potential across fields (Razzaq et al., 2020). This spatial inconsistency results in uneven plant development, asynchronous flowering, and non-uniform crop stands, complicating irrigation, nutrient management, and harvesting operations (Abdullah et al., 2001; Afzal et al., 2012; Irakoze et al., 2020). Moreover, horizontal heterogeneity disrupts source-sink relationships, often causing delayed or incomplete panicle development (Hussain et al., 2018). The field studies reported grain yield losses of 30-50% in heterogeneous saline-sodic soils, primarily due to elevated spikelet sterility, poor grain filling, and irregular reproductive success across the field (Ahmed et al., 2005).

Temporally, fluctuating salinity and sodicity expose rice plants to shifting rather than constant stress, where sudden sodium spikes during microsporogenesis or anthesis are especially damaging, severely impairing floret fertility (Zhao et al., 2020). This spatio-temporal variability induces spikelet sterility through impaired pollen development and anther dehiscence (Kandil et al., 2022). Excessive Na^+^ uptake interferes with sugar and starch metabolism in anthers, resulting in an energy deficit for developing pollen and ultimately producing empty husks despite apparently normal ovule development (Ologundudu et al., 2014). At the same time, photo-assimilate partitioning is severely compromised; saline-sodic stress reduces photosynthetic capacity in the flag leaf, the primary source of remobilized carbohydrates for the developing panicle, while increased energy investment in osmotic adjustment and ion exclusion diverts resources away from grain filling (Hussain et al., 2017). Rice initially responds to patchy stress through root-level avoidance strategy, directing growing toward less hostile zones to enhance water uptake and limit sodium influx, though this plasticity is constrained by the extent of non-toxic soil available (Hasanuzzaman et al., 2009). Additional adaptive mechanisms include osmotic adjustment via compatible solute accumulation, enhanced root system development, and improved ion transport regulation, as shown in **Figure 3** (Osman, 2018). Morphological adjustments such as deeper rooting and modified shoot architecture, such as altered leaf angles and increased thickness, also contribute to survival and improved performance under heterogeneous saline-sodic conditions (Atwell et al., 2014; Chhabra and Chhabra, 2021; Sheoran et al., 2021; Rasheed et al., 2022).

At the cellular level, adaptation relies on membrane transporters, with SOSI pumping Na^+^ out to the soil, HKTI-5 retrieving it from the xylem, and NHX transporters sequestering excess Na^+^ into vacuoles to maintain a favorable K^+^/Na^+^ balance in the panicle and floral organs (Zhang et al., 2015; Osman, 2018). Salt-tolerant rice genotypes, including *Pokkali* and *FL478*, have been shown to selectively exclude Na^+^ while maintaining higher K^+^ levels in their reproductive tissues (Ahmad et al., 2005; De Leon, 2016). Some genotype also adjusts their flowering time to avoid periods of peak stress. Besides, increased expression of vacuolar Na^+^/H^+^ antiporters such as *OsHKTI* allows for the sequestration of toxic sodium ions into vacuoles, protecting cytosolic enzymes (De Leon, 2016; Osman, 2018; Kandil et al., 2022). However, although osmotic adjustment through the accumulation of compatible solutes such as proline, soluble sugars, and glycine betaine helps maintain cell turgor and protect reproductive tissues from dehydration, the high metabolic cost of these processes may limit grain yield (Ahmad et al., 2005; Irakoze et al., 2020). Most agronomic strategies, such as water leaching and amendment applications apply uniformly and fail to address patchy stress, as a result in inefficient resource use and suboptimal yields (Liu et al., 2010). To complement cellular and genotype level mechanisms, foliar-applied humic biostimulants (HB) can enhances root growth, nutrient acquisition, and physiological plasticity (Izquierdo et al., 2024), enabling rice to better exploit microzones with lower stress and maintain yield stability. The key gap lies in understanding rice responses to dynamic, shifting stress conditions rather than a steady-state environment. Addressing this gap advanced soil sensing and high-throughput phenotyping are also required to identify genotypes capable of stable yield formation under dynamic field stress conditions.

## 4 Management strategies for saline-sodic soil heterogeneity

### 4.1 Soil-based interventions

#### 4.1.1 Organic amendments

Organic amendments, derived from natural and waste resources such as farmyard manure, composts, crop residues, manures, and biochar, have been widely used to enhance the physical, chemical, and biological conditions of saline-sodic soils (Elmeknassi et al., 2024). In heterogenetic saline-sodic soils, variability in salinity, sodicity, and alkalinity generates fluctuating ionic stress and unevenly compacted soil layers that hinder root growth, water movement, and infiltration, leads to inconsistent root development and localized stress (Li et al., 2022b). By improving soil structure, nutrient availability, and microbial activity, organic amendments mitigate these complex challenges in rice production (Tejada and Gonzalez, 2006; Safdar et al., 2019). In these stratified soils, organic amendments not only enhance overall soil quality but also buffer variability across patches and depths, helping to stabilize the microenvironments that rice roots experience during sensitive reproductive stages. For instance, biochar has been shown to increase soil organic matter and stimulate microbial activity while simultaneously reducing bulk density and compaction, but its effects are not uniform (Phuong et al., 2020). Li et al. (2022b) reported that biochar enhanced rice root vigor and salt tolerance by lowering malondialdehyde (MDA) levels and improving the Na^+^/K^+^ ratio primarily in surface horizons, whereas deeper sodic layers remained less responsive. Similarly, Jin et al. (2018) found that biochar application improved biomass and yield, but the magnitude of the response varied with subsurface sodicity intensity. Furthermore, the combination of rice husk biochar and organic fertilizer significantly enhanced soil fertility, CEC, and reduced exchangeable Na^+^, although Wijitkosum (2020) reported that these improvements were more pronounced in zones with higher organic carbon accumulation. Besides, Sun et al. (2020) reported that biochar combined with fulvic acid enhanced porosity and water retention, but the magnitude of improvement varied depending on whether soil patches were previously compacted or moderately porous. Zhang et al. (2014) found that biochar with humic acid improved soil properties and crop yield, with greater benefits in surface saline crusts than in deeper sodic horizons. Dos Santos et al. (2021) further demonstrated that biochar derived from corn cobs and sugarcane bagasse improved pore connectivity and water movement, facilitating sodium leaching and reducing EC, SAR, and ESP. However, leaching effectiveness was inconsistent, where compact sodic subsoil layers restricted water flow. Moreover, the microbial dimension of heterogeneity adds further complexity. For example, Elmeknassi et al. (2024) reported that biochar improved microbial biomass and activity in the upper 15 cm of sodic soils but had little effect in subsoil layers, while Smaoui-Jardak et al. (2017) found that organic amendments restored enzymatic activity unevenly across field patches. Jin et al. (2020) found that compost stimulated beneficial microbes in some microsites but not in salt-accumulated patches. However, most studies remain short-term and surface-focused, with limited attention to subsoil processes, interannual variability, or the connections between multi-scale heterogeneity and rice yield.

**Figure 4:**
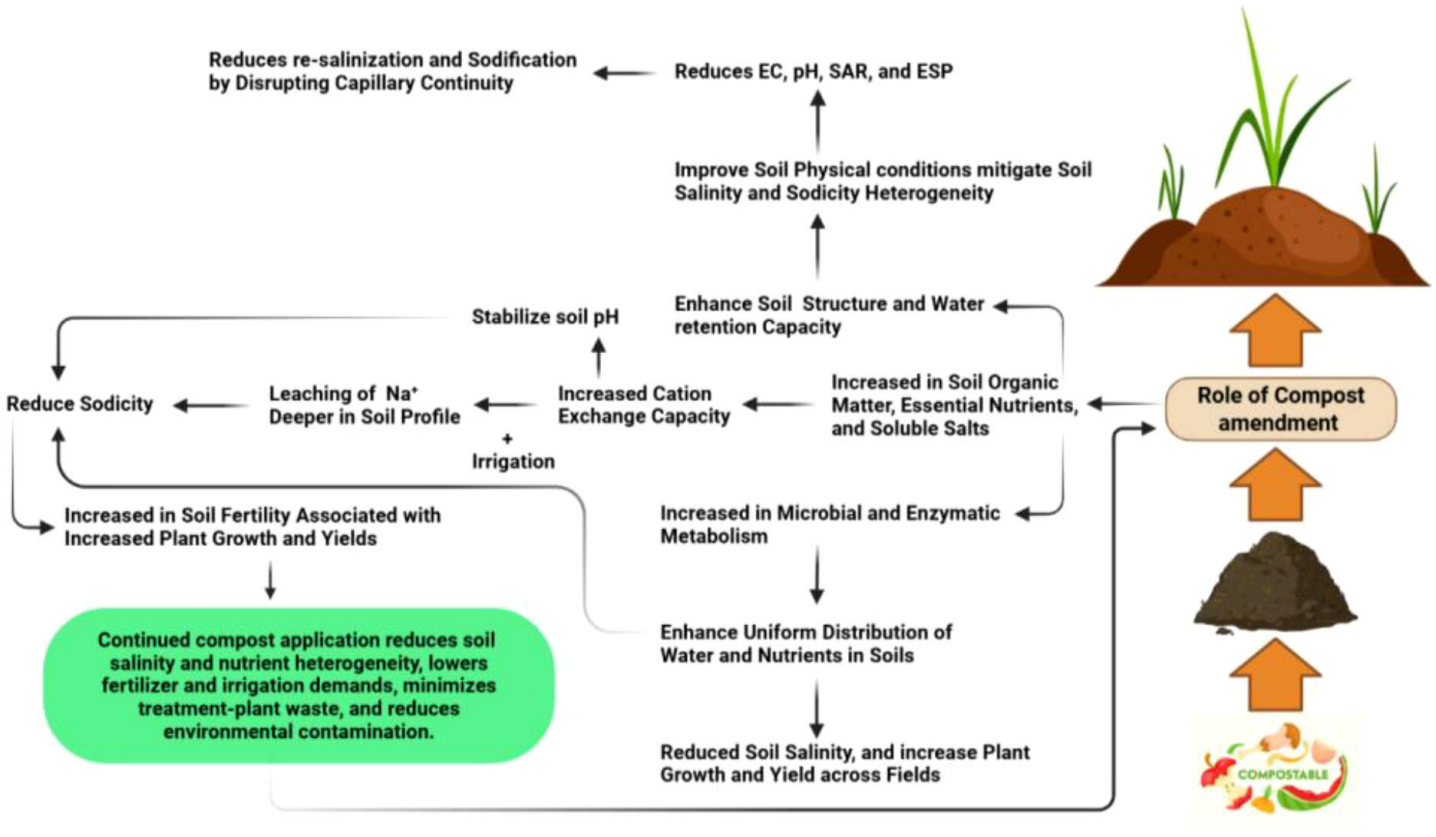
The figure illustrates the mechanisms by which compost ameliorates soil conditions and heterogeneity in salt-affected environments. By increasing organic matter and cation exchange capacity, compost facilitates the leaching of Na^+^ and stabilizes soil pH. These improvements, alongside enhanced water retention and microbial activity, disrupt capillary continuity to prevent re-salinization. Ultimately, the cumulative effect of compost application boosts soil fertility and crop yields while reducing the need for chemical fertilizers and irrigation.

Compost provides important insights into depth- and patch-specific amelioration of saline-sodic soils. For instance, Abdel-Fattah (2012) demonstrated that rice straw compost reduced EC, pH, SAR, and ESP, but improvements were greater in topsoil than subsoil layers. Similarly, Tejada and Gonzalez (2006) reported enhanced aggregation and structure stability following compost application although benefits were concentrated in patches with higher initial microbial activity, while compact sodic zones remained relatively resistant. Vermicompost, rich in Ca²^+^, further contribute to sodicity mitigation by promoting Na^+^ displacement through decomposition-induced acidification of CaCO_3_, thereby reducing ESP (Rezapour et al., 2022). Juleel et al. (2023) reported improved rice yields through humus-soil-root interactions, but both studies highlight site specificity, with greater improvements in friable zones than in sodic hardpans. Luo et al. (2014) demonstrated that compost-biochar mixtures not only improved mean soil properties but also helped create more uniform root-zone conditions. **Figure 4** has illustrated the benefits of compost application, especially saline-sodic soils, and its broader advantages straw incorporation and other organic residues also play key role in managing saline-sodic heterogeneity. Studies by Zhao et al. (2019) and Liao et al. (2020) showed that straw incorporation increased aggregate stability and disrupted capillary continuity, which prevents upward salt movement that typically re-salinizes surface horizons in stratified profiles. This disruption of vertical heterogeneity is critical in paddy systems, where alternating flooding and drying cycles create temporal fluctuations in salinity distribution (Wang et al., 2014). By enhancing porosity and stability, straw incorporation helps dampen these fluctuations and stabilize rice performance (Wijitkosum et al., 2020). Complementing soil-based organic amendments, foliar-applied humic biostimulants provide a plant-cantered approach to mitigate residual heterogeneity by stimulating root growth and nutrient uptake in zones where soil remediation is less effective, thereby strengthening rice performance under spatially variable saline-sodic conditions (Izquierdo et al., 2024). Other organic inputs including green manure, animal manures, whey, fly ash similarly exhibit site-specific benefits, with stronger effects in zones where salts remain mobile compared with structurally rigid sodic layers (Graber et al., 2006; Safdar et al., 2019; Yao et al., 2021).

#### 4.1.2 Inorganic amendments

**Figure 5:**
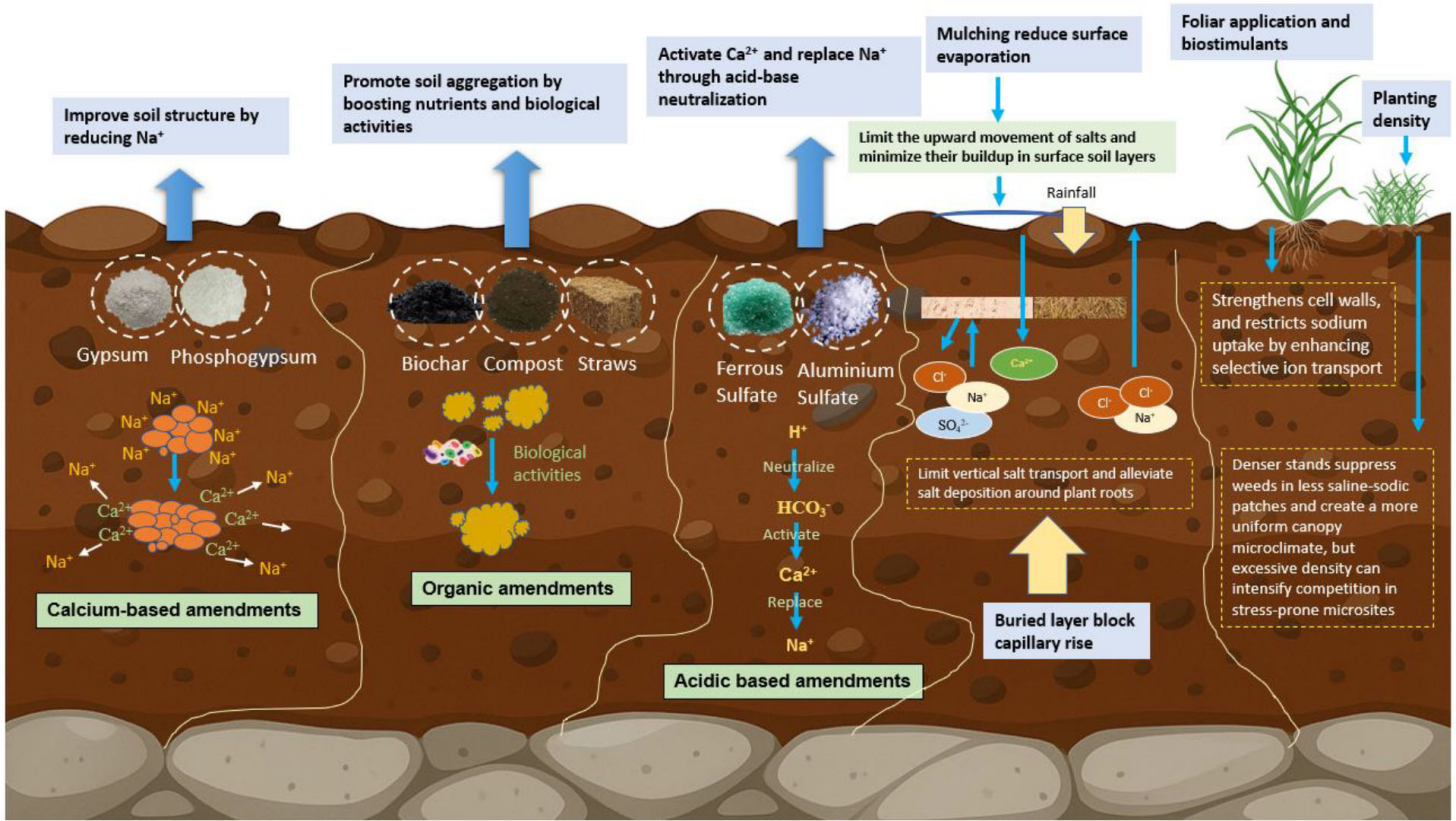
Reclamation strategies of saline-sodic soil heterogeneity through calcium-based amendments (e.g., gypsum, phosphogypsum) improve soil structure by replacing exchangeable Na*^+^* with Ca*^2+^*, while organic inputs (e.g., biochar, compost, crop residues) enhance aggregation, nutrient cycling, and microbial activity. Acidic amendments (e.g., ferrous sulfate, aluminum sulfates) neutralize bicarbonate and mobilize Ca*^2+^*, facilitating sodium displacement. Complementary practices including mulching, subsurface buried layers optimized planting density, and foliar biostimulant applications reduce surface evaporation, block capillary rise, and improve plant resilience.

Inorganic amendments such as gypsum, phosphogypsum (PG), calcium, chloride, calcite, elemental sulphur, and sulfuric acid are widely used to reclaim sodic and saline-sodic soils (**Figure 5**) (Tian et al., 2021). Inorganic amendments mitigate these constraints by modifying ion exchange processes, improving soil structure, and adjusting the chemical environment (Elmeknassi et al., 2024). Gypsum is the most commonly applied amendment because of its cost-effectiveness and high solubility. The Ca^2+^ ions it supplies replace exchangeable Na^+^ on soil colloids, lowering ESP and promoting the flocculation of dispersed clay particles (Abdel-Fattah, 2012). This restoration of soil aggregation reduces bulk density, enhances macroporosity, and improves water infiltration and salt leaching. In the heterogeneity fields, this mechanism is especially critical because sodic hotspots often form impermeable barriers while adjacent zones remain permeable (Rezapour et al., 2022). By stabilizing aggregates and reopening pore networks, gypsum restores hydraulic conductivity across the soil profile, promoting more uniform water movement and alleviating the patchy distribution of water and oxygen to rice roots (Lastiri-Hernandez et al., 2019). Moreover, increasing Ca^2+^ in the soil solution improves the Ca^2+^: Na^+^ ratios, which not only alleviates structural problems but also enhances plant ion uptake by limiting toxic Na^+^ accumulation in rice roots and shoots while promoting the absorption of essential nutrients such as K^+^ and Mg^2+^ (Kim et al., 2016). This is particularly relevant under heterogeneous conditions, where sodic microsites often exhibit nutrient depletion and ionic imbalance, whereas adjacent patches may not; gypsum helps reduce these disparities and ensures a more balanced nutrient supply across the field (Alcívar et al., 2018). The improved electrolyte balance in gypsum-treated soils also stimulates microbial activity, which is typically suppressed in alkaline sodic patches (Nan et al., 2016). This promotes more uniform processes such as carbon mineralization, enzyme activity, and nitrogen cycling, thereby reducing biological heterogeneity and supporting rice tillering and root development (Abdul Qadir et al., 2022).

Other amendments, such as sulfuric acid and elemental sulfur, act through complementary mechanisms that are particularly effective in sodic soils with low calcium carbonate solubility (Ilyas et al., 1993). Sulfuric acid reacts with soil CaCO_3_, releasing Ca^2+^ ions that displace Na+ on exchange sites, a process that is especially valuable in alkaline microsites where pH is high and gypsum dissolution is limited (Abate et al., 2021). Elemental sulphur and pyrite undergo microbial oxidation under flooded conditions, generating *in situ* sulfuric acid, which mobilizes native Ca^2+^ and lowers pH (Abd El-Naby et al., 2019). These reactions are spatially variable but selectively target highly alkaline patches, thereby reducing heterogeneity in soil properties (Abd El-Naby et al., 2019). Similarly, ammonium sulfate contributes to reclamation not only as a nutrient source but also by releasing protons during NH_4_^+^ uptake, which acidifies the rhizosphere and increases Ca^2+^ and Mg^2+^ availability, an advantage in sodic microsites where rice roots face nutrient lock-up (Abdel-Fattah, 2012). Together, these amendments lower ESP, also reducing carbonate and bicarbonate concentrations, thereby decreasing the residual sodium carbonate (RSC) index, a key indicator of sodicity stress in rice paddies.

Among these, PG is a by-product of sulfuric acid and phosphate rock reactions during phosphate production and is widely used as an inorganic amendment (Elmeknassi et al., 2024). It supplies both Ca^2+^ and SO_4_^2-^ ions, which not only replace Na^+^ on exchange sites but also increase ionic strength in the soil solution, as a result suppressing clay dispersion (Tian et al., 2021). PG effectively ameliorates sodicity by supplying Ca^2+^ to replace exchangeable Na^+^, which improves aggregation, enhances salt leaching, modestly lowers soil pH, and ultimately promotes more uniform soil structure and reduces heterogeneity in saline-sodic paddies (Al-Enazy et al., 2018). A field study demonstrates that PG reduces ESP, improves the availability of P and K, and stimulates enzyme activities that regulate microbial communities (Alcívar et al., 2018). In rice paddies, where yield losses often result from uneven panicle development and poor grain filling in sodic-saline microsites, PG application enhances uniformity in yield components including panicle weight and seed-setting rate (Liu et al., 2024). This indicates that PG helps reduce the physiological heterogeneity of rice plants caused by spatial variability in soil constraints (Hasana et al., 2022). Although concerns have been raised about heavy metal accumulation, long-term trials confirmed that PG remains within safe limits when applied responsibly, though continued monitoring is recommended (Duan et al., 2023). The rationale for combining amendments lies in their complementary mechanisms, such as gypsum provides a sustained source of Ca^2+^, sulfuric acid accelerates the dissolution of native CaCO_3_, and organic inputs enhance biological activity and carbon supply (Al-Enazy et al., 2018). For instance, gypsum combined with humic acid not only lowered ESP but also stabilized organic matter-mineral complexes that improved aggregation (Saifullah et al., 2018), while gypsum applied with sulfuric acid increased rice grain yields by 119% under severe sodic conditions (Huang et al., 2022). Such synergies are particularly valuable in heterogeneous fields, where no single amendment can simultaneously resolve structural, chemical, and biological disparities (Rezapour et al., 2022). However, reclaiming heterogenetic saline-sodic soils in rice paddies requires not only selecting appropriate amendments but also understanding their key role in reducing field variability, creating uniform root-zone conditions, and stabilizing yield formation under fluctuating stresses.

#### 4.1.3 Integrated amendment strategies

Organic and inorganic combined amendments are an effective strategy for ameliorating sodic and saline-sodic soils, where heterogeneity in salinity, sodicity, and alkalinity generates microsites of severe stress (Phuong et al., 2020). In contrast to single amendments, mixtures operate through complementary mechanisms e.g., organic inputs such as compost, farmyard manure, crop residues, or biochar enhance soil organic matter, microbial activity, and nutrient cycling, whereas inorganic amendments such as gypsum, PG, calcium chloride, elemental sulfur, or sulfuric acid supply soluble Ca^2+^ or protons that displace exchangeable Na^+^, decrease ESP, and facilitate the leaching of salts from the root zone (**Figure 5**) (Harris and Rengasamy, 2004). This dual action is particularly important in heterogeneous soils, where uneven Na^+^ accumulation causes localized clay dispersion and compaction; the incorporation of organic matter improves aggregation and pore connectivity, enabling Ca^2+^ from PG to move more uniformly through the soil profile and exert de-sodification effects across variable patches (Hasana et al., 2022). When organic matter decomposes, it produces low-molecular-weight organic acids and CO_2_, which acidify the soil microenvironment, solubilize native CaCO_3_, and increase the activity of Ca^2+^ in the soil solution, enhancing the effectiveness of inorganic amendments in displacing Na^+^ (Badia, 2000). In parallel, the organic component improves cation exchange capacity, buffers pH fluctuations, and creates conditions conducive to microbial growth, which accelerates the cycling of C, N, and S and fosters rhizosphere processes that support rice root tolerance under salinity-sodicity stress (Chi et al., 2012). For instance, Mahmoodabadi et al. (2013) demonstrated that applying PG at 30 t ha^-1^ improved rice yield components by reducing ESP and enhancing soil physical properties. Details regarding different soil amendments, their application levels, and their specific effects on reclaiming salt-affected soils are provided in **Table 2**. Similarly, Kim et al. (2016) showed that combining PG with farmyard manure and wood peat further decreased soil pH and EC, increased nutrient availability, and stabilized yields through higher panicle weight and grain filling rates. These studies demonstrate that the interaction between organic and inorganic amendments is synergistic rather than merely additive, as organic matter improves soil structure and microbial activity, facilitating deeper and more uniform penetration of Ca^2+^ and SO_4_^2-^ from PG, while the inorganic component rapidly decreases ESP, creating a more favorable ionic environment for rice roots. Importantly, this synergy helps overcome heterogeneity because in areas where high ESP causes clay dispersion and forms impermeable layers, gypsum or PG alone cannot percolate effectively, and when combined with organic amendments that improve aggregation and porosity, Na^+^ leaching becomes more uniform and spatial variability in sodicity stress is reduced (Elmeknassi et al., 2024). Abdel-Fattah (2012) reported that the application of ammonium sulfate in combination with organic amendments enhanced root-zone acidification, promoted the release of Ca^2+^ and Mg^2+^ from native minerals, and improved nutrient availability in sodic microsites. This process is particularly important in rice paddies, where standing water facilitates the vertical and horizontal redistribution of salts. Such mixtures also help reduce carbonate and bicarbonate concentrations, lower the RSC index, and establish a more favourable ionic balance across the field (Lastiri-Hernandez et al., 2019).

**Table 2:** Soil amendments for amelioration and mitigation of heterogeneity in sodic and saline-sodic soils.

Amendment type	Specific amendments	Effects on soil and crops	Amount/salt type	Crop	References
Organic amendments	Biochar	Improve soil porosity, and reduce Na^+^ accumulation in plant tissues, enhance root growth and yield. Reduces vertical heterogeneity by improving pore connectivity and surface layer structure.	10–40 t ha^-^¹; Saline-sodic soils	Rice, maize, barley	Yao et al., 2021; Luo et al., 2014; Jin et al., 2018
	Compost	Enhances water retention, and nutrient availability, while reduces EC, pH, SAR, ESP and buffers patch-level variability and improve uniformity in root zone conditions.	5–20 t ha^-^¹; Saline, sodic soils	Rice, wheat	Tejada and Gonzalez, 2006; Abdel-Fattah, 2012
	Vermicompost (VC)	Increases soil organic matter, supplies Ca²^+^, reduces ESP. Effect vary with soil friability.	5–15 t ha^-^¹; Sodic soils	Rice, wheat, barley	Rezapour et al., 2022; Juleel et al., 2023
	Straw	Prevents upward migration of salts, increases aggregates, and porosity; buffers vertical heterogeneity during flooding/drying cycles.	4–8 t ha^-^¹; Saline-sodic soils	Wheat, rice	Abdel-Fattah, 2012); Liao et al., 2020; Zhao et al., 2019
	Other Organic Amendments	Includes crop straw, whey, fly ash, farm manure, and green manures. Improve soil structure and nutrient availability.	Varies by type; Saline-sodic soils	Various crop	Graber et al., 2006; Safdar et al., 2019
Inorganic amendments	Gypsum	Enhances nutrient availability, microbial activities, and replaces exchangeable Na^+^ with Ca²^+^, improving soil structure and permeability; Reduces spatial variability of sodicity stress and promotes uniform root-zone conditions.	5–25 t ha^-^¹; Sodic and saline-sodic soils	Wheat, rice	Tian et al., 2021; Nan et al., 2016
	Phosphogypsum (PG)	Enhances nutrient availability, reduce soil pH and ESP. Improve uniformity of soil chemical properties and yield components across heterogenous microsites.	10–30 t ha^-^¹; Sodic soils, saline/sodic soils	Rice, wheat	Hasana et al., 2022; Liu et al., 2010; Huang et al., 2022
	Acidic Fertilizers (e.g., Ammonium Sulphate)	Reduces rhizosphere pH, activates Ca²^+^ and Mg²^+^, and displaces Na^+^; Improves nutrient balance across patches, reducing microsites variability.	100–200 kg ha^-^¹; Saline soils	Various crops	Abdel-Fattah, 2012; Wang et al., 2023
Organic-Inorganic combined	Biochar + Gypsum	Enhances nutrient uptake and improve microbial activity while reduces SAR, ESP, EC and spatial heterogeneity, stabilizing yields.	Biochar: 10–20 t ha^-^¹; Gypsum: 5–15 t ha^-^¹	Rice, wheat	Phuong et al., 2020; Abate et al., 2021
	Compost + Gypsum	Enhance organic matter content, and nutrient availability while reduces soil bulk density and boost crop growth and stabilizing yield across.	Compost: 5–10 t ha^-^¹; Gypsum: 5–15 t ha^-^¹	Rice, wheat	Harris and Rengasamy, 2004; Abdel-Fattah, 2012
	Vermicompost + Gypsum	Boost nutrient uptake and enhance soil structure while reduces Na^+^ content, pH, ESP and spatial heterogeneity.	VC: 5–10 t ha^-^¹; Gypsum: 5–15 t ha^-^¹	Rice, wheat	Elmeknassi et al., 2024; Kim et al., 2016
	Biochar + Compost	Improve wheat yield by reducing SAR, EC, ESP and spatial heterogeneity, stabilizing yields in saline soils.	Biochar: 10–20 t ha^-^¹; Compost: 5–10 L ha^-^¹	Wheat	Abate et al., 2021; Luo et al. (2014)
	Organic + Microbial Inoculation (e.g., PGPR)	Enhances nutrient uptake, improve ionic balance, stimulate enzyme activities, reduce variability in crop response across heterogeneous fields.	PGPR: 10^8^-10^9^ CFU mL^-^¹	Rice Cowpea, Capsicum	Lastiri-Hernandez et al., 2019; AbdEl-Mageed et al., 2020; Sardinha et al., 2003

From a mechanistic perspective, the release of Ca^2+^ from gypsum or PG increases ionic strength in the soil solution, suppressing clay swelling and dispersion, while organic matter-derived polysaccharides act as binding agents, stabilizing aggregates and reducing variability in infiltration rates between different patches of the field (AbdEl-Mageed et al., 2020; Yazdanpanah et al., 2011). Over time, these structural improvements enhance water distribution and support more consistent root proliferation across microsites that would otherwise exhibit strong contrasts in porosity and aeration (Abate et al., 2021). The combined amendments also help in the mitigation of temporal heterogeneity. For example, long-term studies in Northeast China showed that the application of PG combined with organic residues not only reduced soil Na^+^, CO_3_^2-^, HCO^3-^, ESP, but also maintained lower EC and improved microbial biomass. These findings suggest that mixture amendments contribute both to immediate chemical reclamation and to the long-term stabilization of soil function (Phuong et al., 2020). Furthermore, biochar addition in saline-sodic soils has been reported to increase microbial biomass and enzymatic activity in surface layers, but its effect in deeper layers remained limited unless combined with gypsum, which improved percolation and ion exchange throughout the soil profile (Alcívar et al., 2018). This demonstrates that the mixtures of amendments help to overcome the vertical stratification of reclamation effects, resulting in more uniform conditions for rice root systems and reducing the spatial patchiness of stress responses. Similarly, the chemical interactions between organic and inorganic components also play a critical role (Churka et al., 2013). For example, biochar in mixtures can adsorb phytotoxic compounds and salts, reducing their spatial concentration hotspots, while mineral additives like silicon-based amendments can enhance the stability of organic matter, showing its degradation and promoting sustained release of benefits (Nan et al., 2016). Studies in rice paddies have demonstrated that these mixtures not only improve soil health indicators but also mitigate yield variability across heterogeneous fields, with yield increases of 30-56% reported in treated areas compared to untreated controls (Graber et al., 2006). Importantly, such integrated strategies align with sustainability goals, as combining locally available organic residues with moderate rates of gypsum or PG reduces reliance on large single applications of chemical amendments, minimizes environmental risks associated with PG contaminants, and recycles on-farm organic wastes into soil improvement practices (Safdar et al., 2019).

Despite these benefits, relatively few studies have systematically examined the combined use of organic and inorganic amendments in heterogeneous saline-sodic soils. However, organic amendments alone can improve sodic soils; their effects are typically gradual, requiring longer timescales to achieve significant reclamation (Yao et al., 2021). In contrast, inorganic amendments such as gypsum act more rapidly but often lack long-term stability unless supported by organic inputs, as mentioned in **Table 3**. Although the combined use of gypsum, organic matter, and PGP microorganisms has been explored in some contexts, further research is required to clarify their synergistic role in reducing salinity and sodicity stresses under rice-based systems.

**Table 3:** Advantages and limitations of strategies for mitigating heterogeneity in saline-sodic soils in rice systems.

Category	Measures	Application effect	Limitation
Soil-based strategies-Organic amendments	Biochar	Improves soil porosity, reduces bulk density, enhances microbial activity, lowers Na^+^/K^+^ ratio, improves yield and stimulates root vigor.	Effects uneven across depths; limited impact in compact sodic subsoil; short-term focus in most studies.
	Compost	Enhances aggregation, porosity, reduces EC, SAR, ESP; improves nutrient cycling and microbial activity; reduces re-salinization and sodification by disrupting capillary continuity.	Benefits patch-specific (greater in topsoil or biologically active zones); limited effect in sodic hardpans; gradual improvement.
	Vermicompost	Increases organic matter, Ca^2+^ supply, displaces Na^+^, reduces ESP; enhances rice yield via humus-soil-root interactions.	Effects site-specific; higher response in friable zones, less in sodic hard layers.
	Straw incorporation and residues	Prevents upward salt migration, increases aggregate stability, enhances porosity, and buffers vertical heterogeneity during flooding/drying cycles.	Short-term effect; decomposition variability across patches; risk of incomplete uniformity.
	Other organic residues (whey, fly ash, manures, green manures)	Improve soil fertility, structure, and nutrient availability with site-specific benefits.	Variable effectiveness depending on mobility of salts and soil microsites.
Soil-based strategies- Inorganic amendments	Gypsum	Supplies Ca^2+^, replaces exchangeable Na^+^, reduces ESP, restores aggregation, improves infiltration, balances nutrient supply, stimulates microbial activity, stabilizes yield.	Effectiveness limited in soils with low solubility of CaCO_3_; requires monitoring; uneven penetration in highly compacted layers.
	Phosphogypsum (PG)	Provides Ca^2+^ and SO_4_^2-^, reduces ESP and pH, suppresses clay dispersion, enhances nutrient availability, and improves uniformity in yield components.	Risk of heavy metal contaminants; requires safe application rates.
	Sulfuric acid/elemental sulphur	React with CaCO_3_ to release Ca^2+^, reduce PH, mobilize nutrients; effective in alkaline patches.	Site-dependent oxidation; high application cost; potential environmental risks.
	Ammonium sulphate	Lowers rhizosphere pH, displaces Na^+^, enhances Ca^2+^ and Mg^2+^ availability, improves nutrient uptake.	Short-term effect; possible over-acidification in sensitive zones.
Soil-based strategies- Mixture amendments	Organic + Inorganic (e.g., biochar + gypsum, compost + gypsum)	Complementary action: organic matter improves aggregation, microbial activity, and pore continuity; inorganic components supply Ca^2+^/protons for Na^+^ displacement; reduce ESP, SAR, EC, and stabilize yield.	Limited long-term studies; effects vary with site conditions; requires precise rates and integration for sustained impact.
	Organic + microbial inoculants (PGPR, AM fungi)	Enhance nutrient uptake, improve ionic balance, stimulate enzymatic activity, mitigate patch-level stress, and improve root colonization.	Sensitive to environmental conditions; less effective in severely sodic microsites.
Water management practices	Leaching (with/without subsurface drainage)	Removes soluble salts, reduces localized hotspots, improves porosity and root penetration, equalizes moisture distribution.	Effectiveness inconsistent in poorly drained soils; high water demand; risk of waterlogging if mismanaged.
	Flushing	Rapidly reduces surface salinity, homogenizes salt removal in depressions.	Requires abundant water; timing and frequency critical.
	Maintaining standing water	Dilutes salts, prevents upward migration, protects sensitive stages.	Water-demanding; risk of waterlogging under poor drainage.
	Alternate wetting and drying (AWD)	Conserves water, reduces heterogeneity by cyclic flushing and restricting salt rise.	Extended drying can intensify heterogeneity; requires precise timing.
	Precision irrigation (drip, sprinkler)	Supplies water directly to root zone, reduces evaporation-restricting salt crusting, enhances uniformity.	Risk of secondary salinization; higher costs.
	Blending irrigation and fresh water	Prevents excessive accumulation, conserves freshwater, moderates stress in microsites.	Optimal ratios soil- and crop-specific; requires site calibration.
	Mulching and hydrophobic coatings	Reduce evaporation-driven salinity, stabilize soil moisture, reduce micro-hotspots.	Potential environmental issues (plastic mulch); long-term effectiveness uncertain.
Crop management practices	Salt tolerant varieties	Maintain Na^+^/K^+^ balance, vacuolar sequestration, cytosolic protection; stabilize seedling establishment, yield components across heterogeneous fields.	Limited availability; yield potential may lag behind elite varieties.
	Advanced breeding and biotechnology	Improve root foraging, osmotic adjustment, ionic regulation, and yield stability.	Adoption constraints; biosafety and regulatory challenges.
	Optimizes panting schedule	Aligns sowing with low salinity periods, enhances establishment.	Weather- and region- dependent; requires local regulatory challenges.
	Planting density adjustment	Improves canopy microclimate, balances competition, buffers heterogeneity.	Overcrowding increases stress; too sparse reduces yield.
	Integrated nutrient management (INM)	Combines organic and inorganic inputs, improves structure, nutrient cycling, reduces spatial variability.	Requires coordination of resources; site- specific effectiveness.
	Microbial inoculants (PGPR, AM fungi, consortia)	Enhances nutrient uptake, regulate hormones, improve ionic balance, activate stress tolerance pathways.	Sensitive to soil conditions; variable field responses.
	Biostimulants and foliar fertilizers	Stimulate root proliferation, osmoprotectants, antioxidants activity, restrict Na^+^ uptake, maintain ionic balance; mitigate heterogeneity in-season.	Effects short-term; cost and availability constraints.
	Seed priming (KNO_3_, glycine betaine, proline)	Enhances seedling vigor, prepares plants for tolerance to stress.	Benefits diminish over time; requires precise protocols.
	Long rice pasture rotations	Enhanced long-term soil structure, improve organic matter uniformity, and mitigates saline/sodic patchiness through biological cycling.	Required long-term planning; economic trade-offs during pasture phases; site specific livestock management.

### 4.2 Water management as a regulator of soil heterogeneity

Water management plays a pivotal role in rice cultivation, as effective irrigation practices regulate salt distribution patterns and reduce soil heterogeneity (Ezeaku et al., 2015; Choudhary and Mavi, 2019). Among these practices, leaching remains the most effective method for reclamation of saline-sodic soils, as excess irrigation flushes soluble salts from the soil profile, prevents uneven ion accumulation in the root zone, and promotes more uniform plant growth (Yazdanpanah et al., 2011; Liao et al., 2020). This process creates favourable microsites for rice by reducing toxic ion hotspots and balancing nutrient uptake, especially in soils where spatial variability in salinity and sodicity constrains yield (Chi et al., 2012). Zhang et al. (2021) reported that variations in EC, SAR, and ion concentrations in soil leachates under saline-sodic conditions demonstrate that leaching promotes the downward transport and spatial redistribution of soluble salts and exchangeable sodium, which leads to a reduction in the localized zones of severe stress. Similarly, Ghafoor et al. (2012) demonstrated that leaching transformed highly saline-sodic patches into moderately affected zones, improving rice establishment across heterogeneous fields. Moreover, leaching enhances soil porosity and alleviates sodicity-induced compaction, which promotes deeper root penetration and enables rice plants to avoid localized high-sodium pockets while exploiting more uniform soil conditions (Mandal and Raju, 2021). Furthermore, reducing exchangeable Na^+^ through leaching diminishes dispersion heterogeneity, stabilizing soil aggregates and water retention across the field (Liao et al., 2020). When combined with subsurface drainage, leaching is particularly effective in flood-prone regions, as it not only removes salt but also alleviates waterlogging variability that often causes sharp contrasts in rice growth and yield (Mukhopadhyay et al., 2023). In addition, integrating leaching practice with organic amendments such as compost and biochar enhances structural stability and equalizes moisture distribution (Liao et al., 2020). Despite these benefits, the effectiveness of leaching is highly soil-dependent (**Table 3**). For instance, in poorly drained or fine-textured sodic soils, its performance is spatially inconsistent and may even intensify heterogeneity if waterlogging persists in depressions (Choudhary and Mavi, 2019). To mitigate this, subsurface drainage and flushing techniques (periodic ponding followed by rapid discharge of saline surface water) can homogenize salt removal in micro-depressions and prevent re-accumulation at the surface (Mukhopadhyay et al., 2023). Several studies have mentioned that flushing is particularly effective in saline-sodic fields where surface crusting and shallow groundwater contribute to salt accumulation, as it reduces surface salinity more rapidly than conventional leaching and creates more uniform soil conditions (Chi et al., 2012). However, based on this, further research is required to optimize the timing, frequency, and water requirements of flushing under different soil textures and water-limited environments.

Another important strategy is to maintain standing water in rice paddies, which dilutes soluble salts and suppresses their upward movement into the root zone (Riaz et al., 2019). This practice is especially critical during the early growth stages when rice is highly sensitive to spatially variable saline-sodic stress (Siddique et al., 2020). In regions with limited water availability, alternate wetting and drying (AWD) provides a practical compromise. By cyclically flooding and draining the field, AWD can conserve up to 30% of irrigation water while reducing salt heterogeneity in the root zone by flushing salts downward during flooding and restricting their upward movement through capillary rise during drying (Chhabra et al., 2021). Several studies have shown that AWD under moderately saline-sodic conditions improves grain quality and stabilizes yield despite field-scale variability in soil salinity and sodicity (Haque et al., 2021; Nangare et al., 2013). However, the timing of AWD is critical, since extended drying periods may intensify salt redistribution and generate localized salinity-sodicity hotspots that constrain crop performance. Beyond conventional practices, precision irrigation systems such as drip and sprinkler methods can reduce spatial heterogeneity by supplying water directly to the root zone and reducing evaporation-induced salt crusting on the soil surface (Merza et al., 2023). Blending saline with fresh water or applying saline water at alternating intervals has also been shown to moderate heterogeneity by preventing excessive accumulation in sensitive microsites while conserving limited freshwater resources (Nangare et al., 2013). The incorporation of organic amendments such as compost, biochar, or farmyard manure improves soil infiltration and hydraulic conductivity, which promotes more uniform water movement and salt redistribution across saline-sodic fields (Hasana et al., 2022). Likewise, surface mulching and the application of hydrophobic coatings to soils or irrigation lines reduce evaporation-driven salt heterogeneity, maintain more consistent soil moisture, and mitigate micro-scale hotspots of salinity and sodicity (El-Beltagi et al., 2022). Future research should focus on determining optimal blending ratios of saline-sodic and fresh water for different soil types and crops, as well as evaluating the long-term effectiveness of hydrophobic coatings in enhancing soil moisture retention and managing the heterogeneity of salinity-sodicity.

### 4.3 Crop management and agronomic strategies

Crop management practices are a critical strategy for improving sodic and saline-sodic soils in rice systems, as they regulate stress intensity and spatial variability that shape rice establishment, physiological performance, and yield formation (Reddy et al., 2017; Yazhini et al., 2024). Among the available approaches, the development and adaptation of saline-sodic-tolerant rice varieties are crucial, as these cultivars possess intrinsic mechanisms that buffer plants against ionic and osmotic stress (Siddique et al., 2020). Salt-tolerant cultivars such as *Pokkali*, *Nona Bokra*, *FL478*, and others including *IR20*, *MR232*, *BR40*, *CSR89-IR8*, and *Sathra 278* maintain a favorable Na^+^/K^+^ balance through Na^+^ exclusion and vacuolar sequestration alleviating cytosolic toxicity and sustaining photosynthetic efficiency under saline-sodic conditions (Alkahtani et al., 2020). Their strong seedling establishment in highly saline microsites promotes more uniform crop stands, reduces yield penalties associated with patchy crop failure in heterogeneous fields (Korres et al., 2022). Recent advances emphasize root system architecture (RSA) optimization as a complementary strategy for enhancing salinity, sodicity tolerance, particularly under spatially heterogeneity. Improved RSA traits such as deeper rooting increased lateral root density, enhanced aerenchyma formation and steeper root growth angles facilitate access to less saline soil layers, improve water and nutrient acquisition, and limit Na^+^ uptake from highly saline zones (Tariq et al., 2025). These traits are increasingly being targeted through marker-assisted selection and genome-wide association studies, leading to the introgression of loci such as *Saltol* and QTLs including *OsSTL1* and *OsSTL2* into high-yielding backgrounds, thereby stabilizing panicle number and grain filling under saline-sodic conditions (Deng et al., 2011). In parallel, transgenic approaches targeting ion transporters (e.g., *OsHKT1;5*, *OsNHX1*) and transcription factors (e.g., *OsMYB2*) further strengthen ionic homeostasis, while polyploid genotypes such as HN2026-4x exhibit enhanced root plasticity and deeper foraging capacity, enabling plants to exploit less saline soil layers (Kakar et al., 2019). These genetic, biotechnological, and RSA-focused strategies reduce performance variability across heterogeneous saline-sodic soils and support resilient rice production under increasing salinity, sodicity stress (Osman, 2018).

Beyond genetic improvement, the effective and targeted use of fertilizers, amendments, and biostimulants requires accurate diagnosis of plant stress status and spatial variability within fields. Conventional indicators such as leaf chlorosis, growth suppression, and Na^+^/K^+^ ratios provide valuable information but are often insufficient for early-stage stress detection across heterogenous landscapes (Merza et al., 2023). Recent precision crop management approaches integrate remote sensing, proximal sensors unmanned aerial vehicles (UAVs), and GIS-based spatial analysis to enable early identification of salinity, sodicity induced stress before visible symptoms appear (Egea et al., 2026). Spectral indices derived from multispectral and hyperspectral imagery (e.g., NDVI, red-edge indices, thermal signals) have been successfully used to detect spatial patterns of salinity stress, canopy temperature anomalies, and biomass reduction in rice systems (Paliwal et al., 2019; Egea et al., 2026). These diagnostic tools allow field-scale phenotyping of stress responses and provide a decision-support basis for site-specific application of soil amendments, foliar nutrients, and biostimulants.

Optimizing planting schedules further moderates salinity and sodicity effects by aligning rice establishment with periods of lower salt accumulation, typically during rainfall-driven leaching phase or before peak dry-season salinization (Liu et al., 2019). Early planting before seawater intrusion during hydrological flushing improves seedlings establishment, whereas delayed prolongs root exposure to sodium-rich microsites and intensifies yield losses (De Leon, 2016; Schmitz et al., 2022). Studies evidence further suggests that synchronizing growth cycles with natural hydrological flushing moderates patchy salt accumulation, which creates more uniform root-zone conditions that support stable stand development and improved yield formation (Yuan et al., 2020). Planting density also influences heterogeneity management, as denser stands suppress weeds in less saline-sodic patches and create a more uniform canopy microclimate, but excessive density can intensify competition in stress-prone microsites (Yang et al., 2021). In contrast, optimized lower densities in saline-sodic affected fields give plants greater access to water and nutrients while enabling roots to exploit microsites with relatively lower sodicity (Al-Enazy et al., 2018). Beyond adjustment in planting time and density, long-term crop-pasture rotations provide a sustainable strategy for reducing soil heterogeneity. Integrating rice with pasture phases and livestock grazing improves soil structure, enhances biological nitrogen inputs, and gradually ameliorates sodic patches (Lattimore, 1994; Vecchio et al., 2018). This ley-forming approach reduces spatial variability, strengthens ecosystem stability, and lowers long-term dependence on chemical amendments.

Integrated nutrient management (INM) is another useful practice for enhancing rice resilience by improving soil fertility and structure, and microbial functioning, thereby reducing spatial variability (Safdar et al., 2019). INM includes the combined application of organic amendments and inorganic inputs facilitates Na^+^ displacement by Ca²^+^, improves aggregation, and strengthens nutrient cycling as we have mentioned in the above amelioration strategies sections (Elmeknassi et al., 2024). Microbial inoculants, including salt-tolerant PGPR and AM fungi enhances nutrient uptake, regulates hormonal balance via auxin and cytokinin, and activates ACC deaminase pathways that alleviate ethylene-induced stress (AbdEl-Mageed et al., 2020). Synthetic microbial consortia often outperform single inoculants by synergistic interactions (Oo et al., 2015), while AM fungi improve root colonization and stabilize N:P:C stoichiometry, supporting ionic balance, and osmotic adjustment across heterogenous microsites (Yazhini et al., 2024). Biostimulants including seaweed extracts, humic substances, amino acids, and microbial-derived products are increasingly used to complement soil-based strategies, with foliar application enabling rapid uptake by bypassing impaired root-soil interfaces (Kumar et al., 2022). Studies have shown that seaweed extracts stimulate root proliferation, amino acids supply osmoprotectants such as proline, and humic acids enhance antioxidant activity and nutrient chelation (Kapoor et al., 2012; Choudhary and Mavi, 2019). In particular, foliar-applied humic biostimulants (HB) have gained attention as a sustainability-oriented tool, as recent field studies show that HB significantly enhance root system development, nutrient use efficiency, grain yield, and profitability of rice in both saline and non-saline soils (Izquierdo et al., 2024, Izquierdo et al., 2025). Enhanced root growth induced by HB improves soil exploration and access to less saline microsites, which is especially in sodic and saline-sodic soils characterized by strong spatial heterogeneity (Ibraheem et al., 2023). Foliar application of silicon, potassium, and calcium strengthens cell walls, stabilizes plasma membranes, and restricts sodium uptake by enhancing selective ion transport, which alleviates ionic toxicity (Jajoo and Mathur, 2021). Seed priming with agents such as KNO_3_, glycine betaine, or proline further boosts seedling vigor and stress preparedness prior to establishment (Bhantana et al., 2021; Chi et al., 2012). The site-specific application of nutrients and biostimulants supports real-time stress mitigation, directly addressing spatial and temporal heterogeneity during the growth season (Wijitkosum, 2020). Together, with salt-tolerant genotypes, optimized planting practices, and precision-guided management, these integrated approaches strengthen rice resilience and support sustainable reclamation of saline-sodic soils.

## 5 Conclusion and prospective

Sodic and saline-sodic soils pose major challenges to rice production due to excessive sodium, high alkalinity, and degraded soil structure. Moreover, these constraints vary across horizontal, vertical, and temporal scales, creating spatially uneven and dynamically fluctuating stress environments. Despite extensive research, critical gaps remain in understanding and managing field-scale heterogeneity and its interaction with rice growth and yields. Notably, most existing studies relied on uniform soil conditions that fail to capture the field-scale heterogeneity of soil chemical, biological, and structural conditions experienced by rice in real rice fields. In addition, subsoil constraints are poorly explored, despite the key role of deeper horizons in regulating salt storage, drainage, and root penetration. In practice, fields with different levels of salinity or sodicity often receive variable amendment rates but are still managed using uniform fertilizer schedules, irrigation regimes, cultivars, and planting densities. As a result, management efficiency is reduced, and crop performance becomes inconsistent. Furthermore, field operations such as ploughing and land leveling further redistribute salts, mixing severely stressed patches with healthier zones, and generating new heterogeneity that complicates accurate identification and targeted management. Although fields are divided into uniform blocks, no practical or standardized method exists to address within-field spatial variability, limiting the effectiveness of treatments at fine scales. Consequently, traditional uniform management strategies frequently fail in heterogeneous sodic and saline-sodic soils. These limitations extend to current amendment practices, where materials such as PG are applied only pre-transplant, despite salinity and sodicity intensifying during later growth stages. Similarly, organic, inorganic, and combined amendments are usually applied uniformly across inherently heterogeneous fields, resulting in inconsistent outcomes, while tolerant rice cultivars often show unstable performance due to patch-specific stresses. Compounding this problem, large fields lack the machinery to evenly incorporate high amendment doses into deeper layers, resulting in shallow, uneven mixing and lower reclamation efficiency. Additionally, existing amendments need high application rates, making them costly and impractical for large-scale forming. Therefore, there is an urgent need to develop alternative amendments that are effective at lower doses, more affordable, easier to apply, and compatible with site-specific heterogeneous field conditions. Future research should also prioritize multi-location, long-term studies to understand how soil heterogeneity evolves under climate variability and to develop predictive models that integrate spatial and temporal soil dynamics with crop performance. Overall, advancing rice productivity in sodic and saline-sodic soils will require shifting from uniform, surface-level practices toward dynamic, spatially explicit, and mechanistically informed management approaches that consider the full complexity of soil heterogeneity, temporal stress fluctuations, and practical field limitations.
