# Correction: Rice growth and yield formation in heterogeneous sodic and saline-sodic soils: challenges and management strategies

**DOI:** 10.3389/fpls.2026.1837737

**Published:** 2026-04-01

**Authors:** Sulaiman Shah, Zijun Cheng, Xinkai Zhang, Yaseen Khan, Zhengwei Liang, Saira Arshad, Qingxue Meng, Peirou Zhen, Zeao Zhang, Tao Zhang, Jialiang Li, Zhonghua Feng, Muhammad Zahid Mumtaz, Mingming Wang

**Affiliations:** 1 Northeast Institute of Geography and Agroecology, Chinese Academy of Sciences, Changchun, China; 2 Key Laboratory of Vegetation Ecology, Ministry of Education, Jilin Songnen Grassland Ecosystem National Observation and Research Station, Northeast Normal University, Changchun, China; 3 Jilin Da’an Farmland Ecosystem National Field Scientific Observation and Research Station, Da’an, Jilin, China; 4 Center for Eco-Environment Restoration Engineering of Hainan Province, School of Ecology, Hainan University, Haikou, China; 5 China Railway 14th Bureau Group Northwest Engineering Co., Ltd., Xi’an, China; 6 National Key Laboratory of Black Land Protection and Utilization, Northeast Institute of Geography and Agroecology, Chinese Academy of Sciences, Changchun, China; 7 National Saline-Alkali Land Comprehensive Utilization Technology Innovation Center, Northeast Saline-Alkali Land Sub-Center, Da’an, Jilin, China

**Keywords:** growth, *Oryza sativa* L., salinization, sodification, soil heterogeneity, yield

In the published article, there was a mistake in the **Funding** statement. The funding sources “National Key Research and Development Program of China (2022YFD1500502-6)” and “Strategic Priority Research Program of Chinese Academy of Sciences (XDA28110100)” were incorrectly included.

The correct **Funding** statement reads:

“The author(s) declared that financial support was received for this work and/or its publication. This study was financially supported by the Chinese Academy of Sciences; Jilin Province Saline-alkali Land Efficient Management and Comprehensive Utilization of Science and Technology Innovation Major Project (20240203004NC); Special Project for High-tech Industrialization of Scientific and Technological Cooperation between Jilin Province and Chinese Academy of Sciences (2025SYHZ0051).”

The original version of this article has been updated.

